# Type 2 MI induced by a single high dose of isoproterenol in C57BL/6J mice triggers a persistent adaptive immune response against the heart

**DOI:** 10.1111/jcmm.15937

**Published:** 2020-11-29

**Authors:** Elvira Forte, Mona Panahi, Nicoleta Baxan, Fu Siong Ng, Joseph J. Boyle, Jane Branca, Olivia Bedard, Muneer G. Hasham, Lindsay Benson, Sian E. Harding, Nadia Rosenthal, Susanne Sattler

**Affiliations:** ^1^ The Jackson Laboratory Bar Harbor ME USA; ^2^ National Heart and Lung Institute Imperial College London London UK; ^3^ Biological Imaging Centre Central Biomedical Services Imperial College London London UK; ^4^ Central Biomedical Services Imperial College London London UK

**Keywords:** adaptive immune system, auto‐antibodies, autoimmunity, fibrosis, inflammation, isoprenaline, isoproterenol, myocardial infarction, type 2 myocardial infarction

## Abstract

Heart failure is the common final pathway of several cardiovascular conditions and a major cause of morbidity and mortality worldwide. Aberrant activation of the adaptive immune system in response to myocardial necrosis has recently been implicated in the development of heart failure. The ß‐adrenergic agonist isoproterenol hydrochloride is used for its cardiac effects in a variety of different dosing regimens with high doses causing acute cardiomyocyte necrosis. To assess whether isoproterenol‐induced cardiomyocyte necrosis triggers an adaptive immune response against the heart, we treated C57BL/6J mice with a single intraperitoneal injection of isoproterenol. We confirmed tissue damage reminiscent of human type 2 myocardial infarction. This is followed by an adaptive immune response targeting the heart as demonstrated by the activation of T cells, the presence of anti‐heart auto‐antibodies in the serum as late as 12 weeks after initial challenge and IgG deposition in the myocardium. All of these are hallmark signs of an established autoimmune response. Adoptive transfer of splenocytes from isoproterenol‐treated mice induces left ventricular dilation and impairs cardiac function in healthy recipients. In summary, a single administration of a high dose of isoproterenol is a suitable high‐throughput model for future studies of the pathological mechanisms of anti‐heart autoimmunity and to test potential immunomodulatory therapeutic approaches.

## 1 INTRODUCTION

Type 2 myocardial infarction (T2MI) is a heterogeneous syndrome resulting in ischaemic damage to the heart because of insufficient myocardial oxygen supply.^1^, ^2^ Unlike type 1 MI (T1MI), T2MI occurs in the absence of coronary artery disease (CAD) or the disruption of an atherosclerotic plaque. Instead, other conditions including hypoxaemia, hypo/hypertension, tachycardia or tachyarrhythmias lead to an imbalance between myocardial oxygen supply and demand, culminating in cardiomyocyte necrosis.^3^, ^4^ Importantly, T2MI is still an under‐recognised clinical entity, despite a recent estimate that 58% of total MI patients in fact suffer from T2MI.^5^ The development of high sensitivity biomarker assays for cardiac troponin I, which allow the detection of even mildly elevated levels of cardiac troponin in the absence of CAD or a T1MI diagnosis, has made the discrimination between T1MI and T2MI possible.^6^ T2MI patients face poor short‐ and long‐term outcomes and a heart failure risk equal to T1MI.^7^, ^8^ Heart failure, the final common pathway of a range of cardiovascular conditions, affects up to 62.7% of MI patients within 6 years after infarction.^9^

Myocardial necrosis triggers an immediate response by the innate immune system which is crucial for quick tissue repair.^10^ However, excessive inflammation is a potent pathological factor in heart failure and has been studied extensively.^11^, ^12^, ^13^ Recently, it has also been suggested that the adaptive immune system is activated after MI and may play a role in the deterioration of heart function and the development towards heart failure.^14^, ^15^, ^16^, ^17^ However, pathological mechanisms are far from understanding and there is an urgent need for better recognition and understanding of these immunological processes, if they are to become the target of future therapeutic applications.

Investigation of the adaptive immune response to cardiac damage is highly complex and still relies heavily on in vivo experimentation. In rodents, surgical coronary artery ligation of the left anterior descending artery (LAD) is the most commonly used experimental model for human T1MI,^18^ whereas a dedicated model for T2MI is missing. The use of the ß‐adrenergic agonist isoproterenol to induce cardiomyocyte necrosis has been described over 50 years ago in rats ^19^, ^20^ and has been characterised extensively.^21^, ^22^ It now routinely serves as a long‐term low‐dose treatment to induce cardiac hypertrophy,^23^, ^24^, ^25^ and few research groups have also used it to induce acute infarct‐like necrotic lesions by injection of high doses.^26^, ^27^, ^28^ Importantly, the isoproterenol model recapitulates several of the salient features of T2MI. The acute ß‐adrenergic activation has a strong positive inotropic and chronotropic effect,^29^ leading to increased oxygen demand, dysregulation of ryanodine receptor 2 (RyR2) and consequent intracellular calcium leakage.^27^ These are all factors that contribute to cardiomyocytes necrosis and acute myocardial injury.

Here, we characterise the effects of a single high dose of isoproterenol on the adaptive immune response to ischaemic myocardial damage in C57BL/6J mice. We confirm that isoproterenol‐induced myocardial necrosis is severe enough to trigger immune activation and replacement fibrosis. We show that this cardiac injury activates CD4+ helper T cells and subsequently induces persistent IgG+ anti‐heart auto‐antibody production. A single high dose of isoproterenol is therefore a simple, resource‐efficient and minimally invasive model of T2MI to investigate the role of post‐MI anti‐heart autoimmunity.

## 2 MATERIALS AND METHODS

Mice: All animal procedures carried out at Imperial College London were approved by the Imperial College Governance Board for Animal Research and in accordance with the UK Home Office Animals (Scientific Procedures) Act 1986 and Directive 2010/63/EU of the European Parliament on the protection of animals used for scientific purposes. All animal works carried out at The Jackson Laboratories were approved by The Jackson Laboratory Institutional Animal Care and Use Committee and were in accordance with national and international regulations. Mice used were 8‐ to 12‐week‐old male or female C57BL/6J mice; within each experiment, experimental groups were age‐ and sex‐matched. Mice were purchased from Charles River UK (Imperial College London) or bred in house (The Jackson Laboratories). After random allocation to experimental groups, mice were housed under SPF conditions (Imperial College London) or in conventional cages (The Jackson Laboratories) in temperature‐controlled facilities on a 12‐h light/dark cycle on standard diet.

Isoproterenol treatment and tissue harvest: Mice were treated with isoproterenol HCL (Sigma‐Aldrich, St. Louis, MO, USA) in Dulbecco's phosphate‐buffered saline (DPBS; Sigma‐Aldrich) by a single intraperitoneal injection of 160 mg/kg. Control mice were treated with DPBS at equivalent volume. On the day of tissue collection, 200 μl blood was collected. Blood was incubated on ice for 30 minutes and then centrifuged for 3 minutes at max rcf for serum collection. Organs were isolated after *in situ* perfusion with ice‐cold DPBS supplemented with 0.9 mM CaCl_2_ through the apex of the left ventricle of the heart to clear blood from heart chambers and blood vessels.

Adoptive transfer: Donor mice were treated with isoproterenol HCL (Sigma‐Aldrich, St. Louis, MO, USA) in DPBS by a single intraperitoneal injection of 160 mg/kg. 4 weeks after isoproterenol injection, serum and spleens were harvested. Excised spleens were mashed through a 70μm cell strainer to generate single‐cell suspensions, followed by red blood cell lysis using Red Blood Cell Lysis Buffer (Sigma‐Aldrich, St. Louis, MO, USA). CD4^+^ T cells were enriched using the EasySep™ Mouse CD4^+^ T Cell Isolation Kit (STEMCELL Technologies, Cambridge, UK) as per the manufacturer's instructions. Recipient mice were pre‐treated 24h before adoptive transfer with a single topical application to the right ear of 3.3μg/μl/g bodyweight (in 1:3 ethanol:acetone) of Resiquimod (Sigma‐Aldrich, Dorset, UK). Recipients received a single intravenous injection of either PBS, 3x10^7^ control splenocytes, 3x10^7^ isoproterenol splenocytes, 1x10^7^ CD4^+^ T cells or twice‐weekly intraperitoneal injection of 200ul isoproterenol serum for 2 weeks, respectively. Pathological effects of adoptive transfers were analysed after 4 weeks.

Cardiac magnetic resonance imaging (MRI): Longitudinal cardiac MRI was performed before and 2 weeks post‐isoproterenol injection. Mice were anaesthetised with 1%‐2.5% isoflurane adjusted to maintain the respiratory rate of 65‐75 breaths/min. Body temperature was maintained at 37 ± 0.5°C by a heating mat. Respiration, ECG and body temperature were continuously monitored (SA Instruments, Stony Brook, NY, USA). MRI was performed on a 9.4 T‐BioSpec system (Bruker BioSpin, Ettlingen, Germany) equipped with a mouse heart array receiver. For localisation of the heart, low‐resolution ECG and respiratory triggered gradient‐echo scout scans were initially acquired in axial, sagittal and coronal orientations followed by highly resolved vertical long‐axis (VLA) and horizontal long‐axis (HLA) views. Ejection fraction (EF), end‐diastolic volume (EDV), end‐systolic volume (ESV) and mass were quantified from a multi‐slice multi‐frame CINE sequence with slices oriented in short axis covering the entire left ventricle (LV), making sure to include the mitral annulus and the apex. Acquisition parameters are as follows: repetition time (TR)=RR interval/number of frames (∼10 ms for 11 frames), TR_effective_ = RR interval, echo time (TE) = 2.05 ms, flip angle = 25°, slice thickness = 1 mm (continuous slices), acquisition matrix = 122 × 122 and field of view = 20 × 20 mm^2^, leading to a spatial in‐plane resolution of 164 × 164 μm^2^.

Echocardiography: The high‐frequency ultrasound system Vevo 770 (VisualSonics Inc, Toronto, Canada) with a 30‐MHz linear transducer was used for transthoracic echocardiography. Anaesthesia was induced at 1%‐2% isoflurane, and warmed ultrasound gel and a heating pad were used to maintain body temperature at 37 ± 0.5°C and heart rate at 450 ± 50 beats per minute. Left ventricular (LV) parameters were calculated from M‐mode traces at base, mid‐level and apex in parasternal long‐ and short‐axis views. Analysis was performed using the Vevo 770 workstation software.

Histology and scoring of damage parameters: Organs were excised as described above, fixed in 10% neutral buffered formalin overnight and stored in 70% ethanol. For wax‐embedding and histology, tissue samples were dehydrated in an increasing gradient of ethanol and embedded in paraffin. Five‐µm sections were cut and de‐waxed and rehydrated in an ethanol gradient. Sections were stained with haematoxylin and eosin (H&E) and Picrosirius Red. All reagents were purchased from Sigma‐Aldrich (Sigma‐Aldrich, Dorset, UK). Semi‐quantitative scoring of heart sections was performed as established previously.^30^ H&E‐stained sections were used to analyse and score immunopathology. Picrosirius Red staining was used to analyse and score fibrosis. Individual parameters were scored on a scale of 0 (none), 1 (mild), 2 (moderate) to 3 (severe). Scores were obtained from 4 areas each on 2 midline cross sections per animal by a blinded researcher. Images were captured using a Hamamatsu NanoZoomer 2.0 slide scanner (Hamamatsu, San Jose, CA, US) and a LMD7000 microscope (Leica microsystems, Milton Keynes, UK) and processed for quantification of nuclei (cell count) and area of fibrosis using NDP.view2 Plus Image viewing software (Hamamatsu, San Jose, CA, US) and the public domain software ImageJ (NIH; http://rsb.info.nih.gov).^31^

Cardiac troponin I and anti‐heart auto‐antibody ELISA: A mouse cardiac troponin I (cTnI) ELISA Kit (MyBioSource, San Diego, CA, USA) was used to determine cTnI concentrations in post‐isoproterenol serum as instructed by the manufacturer. Serum samples were diluted two‐fold in supplied diluent. A standard curve was generated to calculate cTnI concentrations in pg/ml. The ELISA protocol for the detection of mouse anti‐heart auto‐antibodies was optimised as described previously.^30^ ELISA plates (SpectraMax Paradigm, Molecular Devices, UK) were coated with 50µl per well of 4µg/µl pig heart lysate (Novus Biologicals, Bio‐Techne, Abingdon, UK) diluted in PBS overnight at 4 ºC. Plates were washed 3 times for 5 minutes each with 200 µl per well of ELISA washing buffer. 50 µl of post‐isoproterenol serum was added diluted 1:10 and 1:100 for overnight incubation at 4 ºC. Detection reagents were anti‐mouse IgG‐HRP (all from BioLegend, London, UK). Optical densities were measured at 450 nm using a SpectraMAX i3 Microplate Reader (Molecular Devices, San Jose, CA, USA).

Heart and lymph node single‐cell preparation: To generate single‐cell cardiac suspensions for flow cytometry, a modified digestion protocol was used as previously described.^32^ Hearts were excised, chopped 30 times with surgical scissors and placed in 3 ml digestion buffer. The digestion buffer consisted of 2 mg/ml collagenase type IV (Worthington Biochemical Corporation, Lakewood, NJ, USA), 1.2 U/ml dispase II (Thermo Fisher Scientific, Waltham, MA, USA) and 16.6 ul/ml precision count beads (BioLegend, San Diego, CA, USA) in DPBS supplemented with 0.9 mM CaCl2. The tissue was incubated at 37ºC for 15 minutes. After incubation, tissue was triturated 12 times. Tissue was incubated and triturated 2 more times for a total of 45 minutes. The final trituration was increased to 30. Tissue was filtered through sterile cheesecloth and washed with ice‐cold DPBS supplemented with 0.9 mM CaCl2 for 20 minutes at 200 rcf at 4 ºC. Lymph nodes were incubated at 37ºC for 30 minutes in 3 ml and 0.5 ml digestion buffer, respectively. Lymphoid digestion buffer consisted of 0.24 U/mg 400 Mandl U/ml collagenase D (Roche Diagnostics GmbH Mannheim, Germany) in FBS‐free DMEM. Cells were washed twice in FACS buffer supplemented with 2% FBS spun for 5 minutes at 400 rcf at 4 ºC.

Flow cytometry: Single‐cell preparations were used for flow cytometric analysis. Antibodies used were as follows: anti‐mouse CD45 (CD45‐APC‐Cy7—cat. 103116, clone 30‐F11, LOT B185138, dilution 1:800), anti‐mouse CD3 (CD3‐APC—cat. 100235, clone 17A2, LOT B166471, dilution 1:200), anti‐mouse CD4 (CD4‐FITC—cat. 115505, clone 6D5, LOT B131781, dilution 1:200), anti‐mouse CD8a (CD8a‐PE—cat. 101207, clone M1/70, LOT B166034, dilution 1:400), anti‐mouse CD62L (CD62L‐AP—cat. 137607, clone 29A1.4, LOT B152186, dilution 1:200) and anti‐mouse CD44 (CD44‐APC—cat. 137607, clone 29A1.4, LOT B152186, dilution 1:200). All were purchased from BioLegend (BioLegend, London, UK). Antibody dilutions in cell staining buffer containing 1% TruStain fcX™ (antimouse CD16/32) antibody (both from BioLegend, London, UK) were used to stain according to the manufacturer's protocol. Samples were acquired using a BD LSRII (Becton Dickinson, Oxford, UK) and analysed using FlowJo version 10.6.1 software (Tree Star, Ashland, OR, USA).^31^

Immunohistochemistry: For the detection of in vivo antibody deposition in cardiac tissue, 5‐µm sections of frozen post‐isoproterenol hearts were stained with goat anti‐mouse IgG‐FITC (cat. F5387, Sigma‐Aldrich, Dorset, UK) and rat anti‐mouse IgM‐PE (cat. 406507, BioLegend, London, UK). Images were captured using a LMD7000 microscope (Leica microsystems, Milton Keynes, UK) and processed using the public domain software ImageJ (NIH; http://rsb.info.nih.gov).

Experimental planning and statistical analysis: Individual animals were randomly allocated to experimental groups from a homogeneous age‐ and sex‐matched pool, husbandry and care was provided by blinded staff, and analysis was performed by a blinded researcher. Initial exploratory titration experiments used n = 3 for welfare reasons. Subsequent experimental group sizes were defined based on the effect size of 3.8 achieved in infiltration scoring experiments. To achieve 95% power, with 0.05 set as alpha error, a group size of 3 is necessary for the primary readouts of infiltration. Smaller effect sizes were expected and accepted as biologically significant for flow cytometry, MRI and ELISA results; thus, group sizes were increased for these experiments. Statistical analysis was performed using GraphPad Prism 8, and data were presented as mean ± s.e.m throughout. Normal distribution of parametric data was tested using Shapiro‐Wilk test. Comparison between 2 groups was performed using Student's *t* test with Welch's correction to account for possible differences in variation between control and treatment group. Comparison between multiple experimental groups was performed using one‐ or two‐way ANOVA with Dunnett's multiple comparisons post hoc test to obtain multiplicity‐adjusted p‐values. Scoring data were analysed using non‐parametric Kruskal‐Wallis test with Dunn's multiple comparisons post hoc test to obtain multiplicity‐adjusted p‐values. Differences were considered significant at *P* < .05.

## 3 RESULTS

### 3.1 A single dose of isoproterenol induces cardiac histopathology reminiscent of human type‐2 MI in C57BL/6J mice

**Figure 1:**
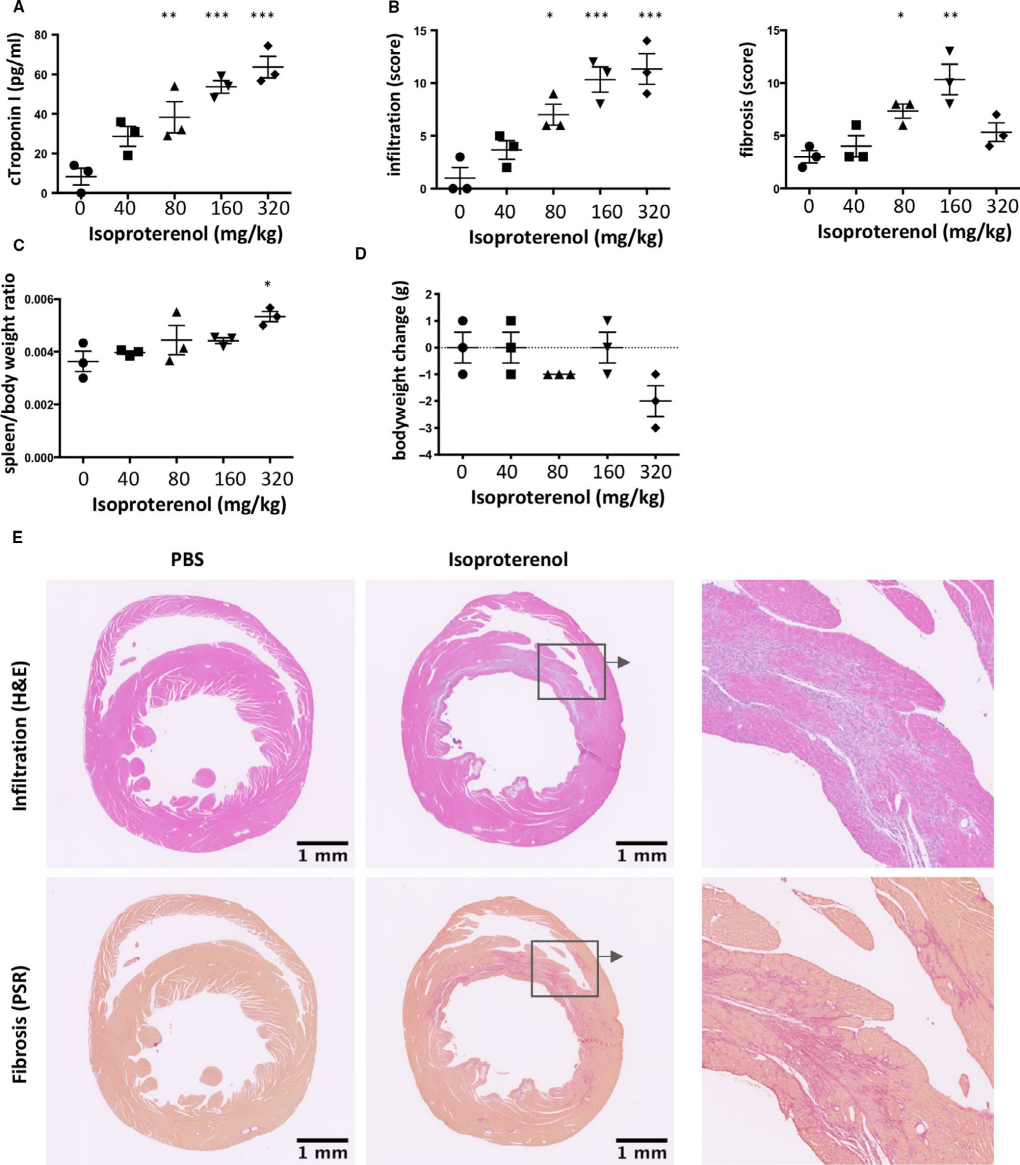
A single high dose of isoproterenol induces myocardial inflammation and fibrosis in C57BL/6J mice. Male C57BL/6J mice were treated with a single increasing dose of isoproterenol to induce myocardial ischaemia and cardiomyocyte necrosis and analysed 2 weeks after challenge. A, Serum cardiac troponin I (cTroponin I) levels to confirm myocardial injury. B, Infiltration and fibrosis were scored on a scale from 0 to 3 (none, mild, moderate, severe) in 4 fields of view in 2 heart cross sections at mid‐level per mouse. C, Splenomegaly (spleen/bodyweight ratio) as a measure of systemic immune activation. D, Change in bodyweight. E, Micrographs of H&E‐ and Picrosirius Red–stained paraffin‐embedded cross sections of the hearts showing mononuclear cell infiltration (H&E) and interstitial fibrosis (Picrosirius Red). Data are expressed as mean ± s.e.m., *P < .05, **P < .001, ***P < .0001 (B: non‐parametric Kruskal‐Wallis with Dunn's multiple comparisons post hoc test comparing each time‐point to baseline. A, C and D: one‐way ANOVA with Dunnett's multiple comparisons post hoc test comparing each time‐point to baseline)

Isoproterenol induces cardiomyocyte necrosis with necrotic cardiomyocytes mostly localised in the sub‐endocardium.^33^ Different mouse strains show variable susceptibility to induction of cardiac damage, and C57BL/6J mice have a robust cardiac phenotype relatively resistant to myocardial fibrosis.^34^, ^35^, ^36^ They are however among the best characterised mouse strains and commonly used for genetic modification and immunological studies. To allow subsequent use of this common mouse strain, we performed a dose titration experiment to investigate whether C57BL/6J mice were susceptible to developing cardiac lesions while maintaining high animal welfare standards. Male C57BL/6J mice were treated with a single bolus intra‐peritoneal injection of increasing doses of isoproterenol starting with 40mg/kg up to 160mg/kg. Isoproterenol effects were evident from 15 minutes after injection, when mice ceased moving and increased respiration rates were observed. Most mice recovered within a few hours as judged by the return of normal feeding and grooming behaviour and were expected to not show any more clinical signs of treatment after 24h. Mortality was rare (cumulative mortality over a year was estimated to be below 1%), occurs within the first day and is presumably because of acute arrhythmic problems. 80 and 160mg/kg isoproterenol induced significant increase in cardiac troponin I (cTnI) in the serum 24h after injection (Figure 1A). cTnI is the current gold standard clinical biomarker for myocardial necrosis and allows detection of T2MI in the absence of CAD.^6^, ^37^

80 and 160mg/kg isoproterenol also increased myocardial infiltration and fibrosis 2 weeks after injection (Figure 1B). Spleen/bodyweight ratio as a measure of systemic inflammation and bodyweight as indicator of overall health were not affected with doses up to 160mg/kg (Figure 1C, D). 160mg/kg each on 2 subsequent days however increased spleen/bodyweight ratio and reduced bodyweight. A single injection of 160mg/kg was thus chosen as standard dose for subsequent experiments. Histopathological analysis of the hearts showed tissue damage resulting in granulation tissue and fibrosis. Consistent with catecholaminergic damage, injury was most prominent in the sub‐endocardium (Figure 1E).

### 3.2 Off‐target histopathology in kidney, liver, lung and skeletal muscle is negligible

**Figure 2:**
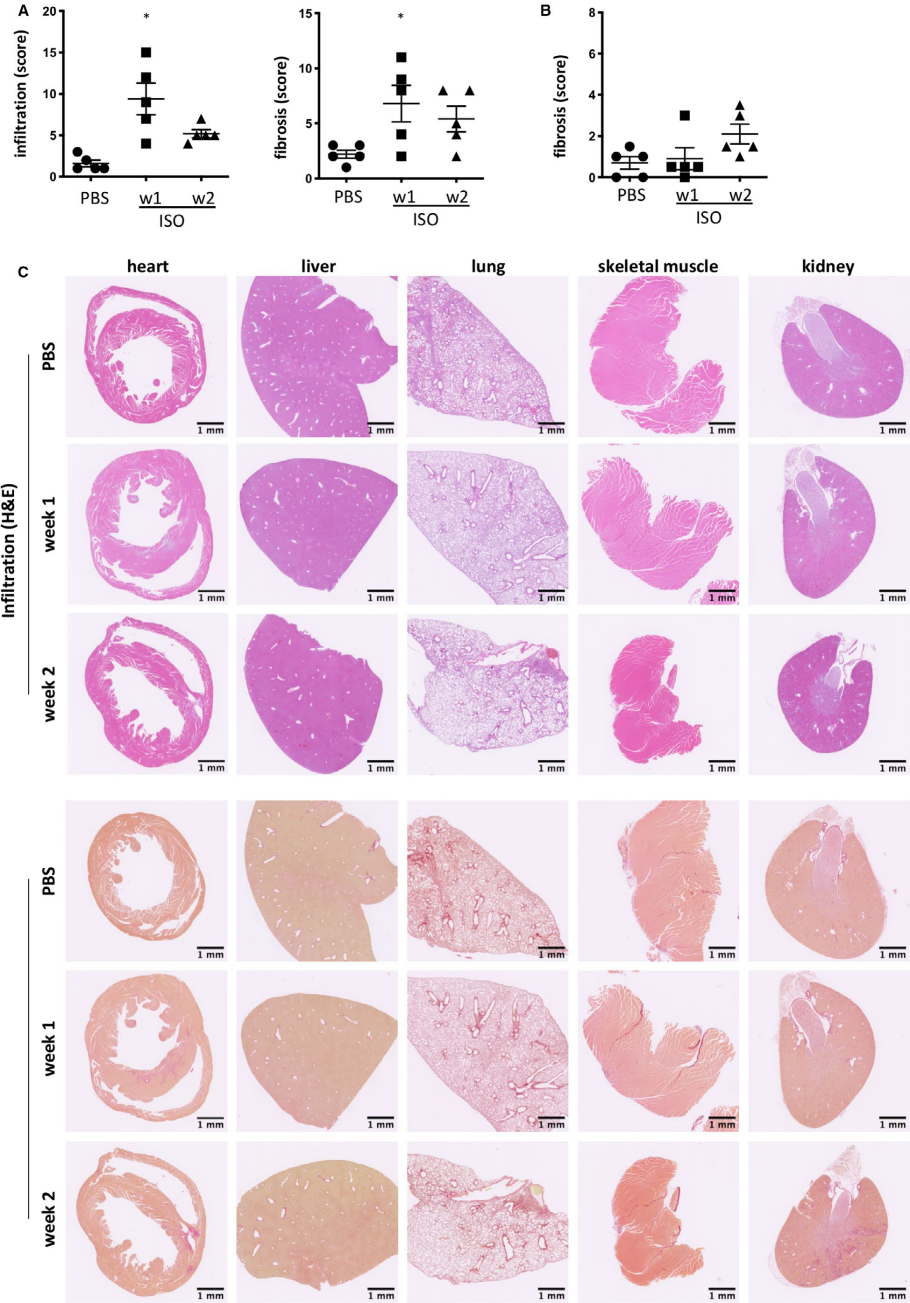
A single high dose of isoproterenol does not affect liver, lung and skeletal muscle, but pathological changes in the kidneys are observed secondary to cardiac damage at week 2. Male C57BL/6J mice were treated with a single dose of isoproterenol to induce myocardial ischaemia and cardiomyocyte necrosis. A, Myocardial infiltration and fibrosis were scored on a scale from 0 to 3 (none, mild, moderate, severe) in 4 fields of view in 2 heart cross sections at mid‐level per mouse. B, Kidney fibrosis was scored on a scale from 0 to 3 (none, mild, moderate, severe) in 4 fields of view in 2 cross sections per mouse. n = 5/group, symbols represent individual mice. Data are expressed as mean ± s.e.m., *P < .05 (non‐parametric Kruskal‐Wallis with Dunn's multiple comparisons post hoc test comparing each time‐point to baseline). C, Micrographs of H&E‐ and Picrosirius Red–stained paraffin‐embedded sections of post‐isoproterenol organs for assessment of mononuclear cell infiltration (H&E) and interstitial fibrosis (Picrosirius Red). Representative image shown

To define the degree of off‐target damage to other organs, we performed a thorough histopathological assessment of kidneys, liver, lung and skeletal muscle at weeks 1 and 2 after injection of 160mg/kg isoproterenol. Myocardial infiltration and fibrosis were confirmed (Figure 2A). No pathological changes were observed in the other tested organs in any of the mice (n = 5/group) at week 1 (Figure 2B, C, S1). Although isoproterenol has been shown previously to induce acute necrosis in skeletal myocytes,^38^ the regenerative ability of skeletal muscle ^39^ appears to allow it to restore healthy morphology by week 1. At week 2, mild acute tubular injury and fibrosis were detectable in the kidneys (Figure 2B, C, S1). This thus happened after established cardiac damage. Renal damage has been documented as secondary to cardiac damage after experimental surgical LAD ligation ^40^ and in human MI patients.^41^ The observed pattern of renal injury and fibrosis was therefore broadly consistent with the established concept of cardiorenal syndrome, which is renal damage/dysfunction due to cardiac damage/dysfunction, and vice versa.^42^

### 3.3 A single dose of 160mg/kg isoproterenol induces cardiac dilation and impairs systolic function in C57BL/6J mice

**Figure 3:**
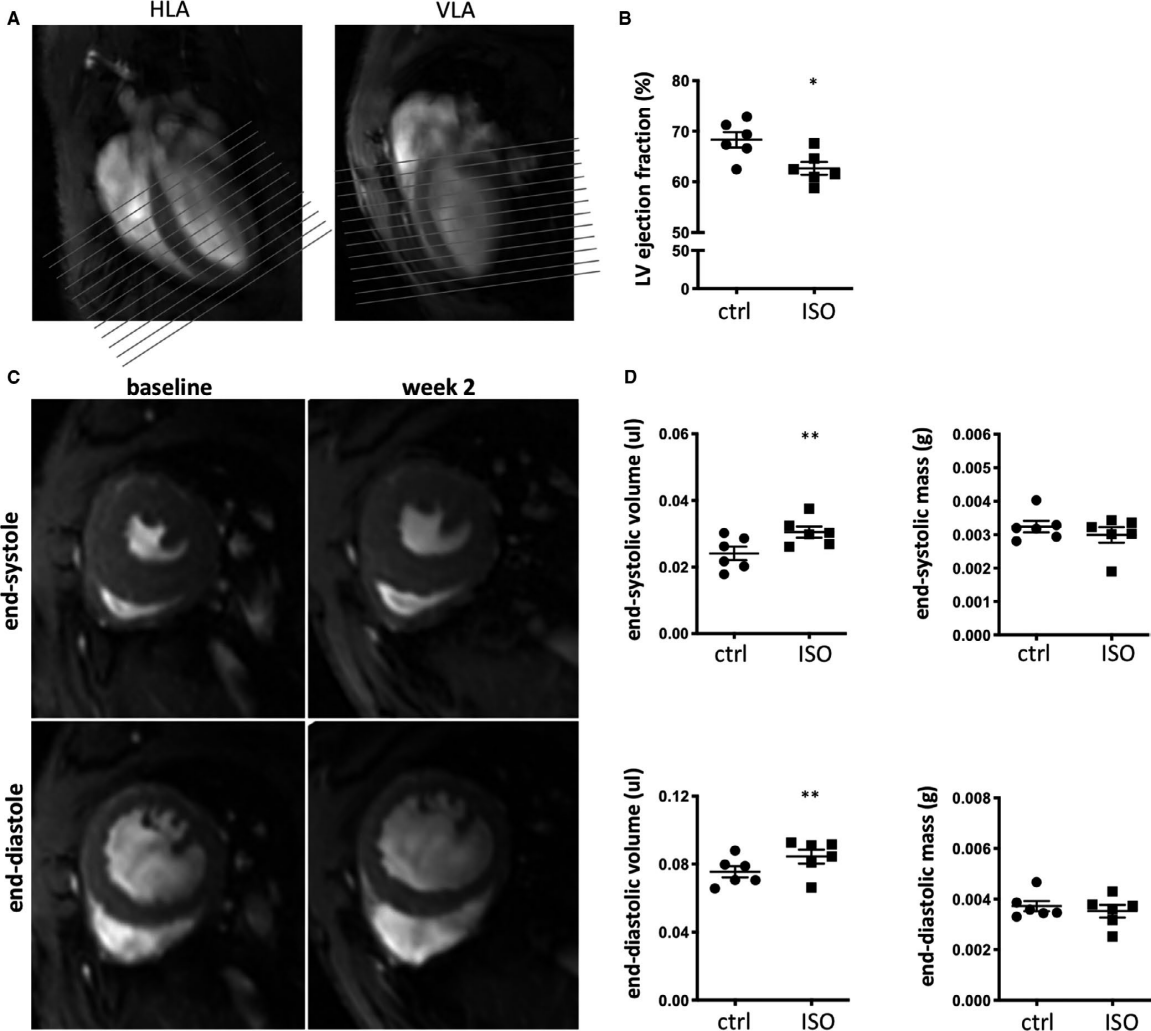
Isoproterenol treatment causes significant volumetric changes in the heart and impaired systolic function. Male C57BL/6J mice were treated with a single dose of isoproterenol and analysed for cardiac morphology and function before and 2 weeks after isoproterenol challenge. A, Representative long‐axis views (HLA and VLA) required for accurate planning of multi‐stack short‐axis CINE slices (grey lines). Each cardiac image was segmented at end diastole and end systole from base to apex to derive the outer and inner border of the left ventricle (LV) required for LV end‐diastolic volume (EDV), LV end‐systolic volume (ESV), LV ejection fraction (EF) and LV mass assessment. B, Quantification of LV EF pre‐ and 2 weeks post‐isoproterenol administration. C, Representative images of end‐systolic and end‐diastolic hearts pre‐ and 2 weeks post‐isoproterenol administration D, Quantification of LV EDV, ESV and mass pre‐ and 2 weeks post‐isoproterenol administration. n = 6/group, symbols represent individual mice before and after isoproterenol treatment. Values represent mean ± s.e.m.; *P < .05, **P < .005, one‐tailed, paired Student's t test

To assess whether immunopathology observed by histology was severe enough to affect cardiac morphology and function, we performed cardiac magnetic resonance (CMR) imaging before and 2 weeks after isoproterenol injection. Previous studies using variable dosing regimens reported morphological changes and diastolic dysfunction.^24^, ^34^, ^43^ After planning of vertical long‐axis (VLA) and horizontal long‐axis (HLA) cardiac views (Figure 3A), the endocardial and epicardial borders were contoured from apex to base at end‐diastole and end‐systole phases (Figure 3C). A mild but significant drop in left ventricular (LV) ejection fraction (Figure 3B) was detectable 2 weeks after isoproterenol treatment. This was paralleled by an increase in LV end‐diastolic and end‐systolic volumes (Figure 3D) suggestive of LV dilation. Isoproterenol administration did not cause significant alteration of LV mass by week 2 after treatment (Figure 3D).

### 3.4 Model refinement and reduction in animal numbers

**Figure 4:**
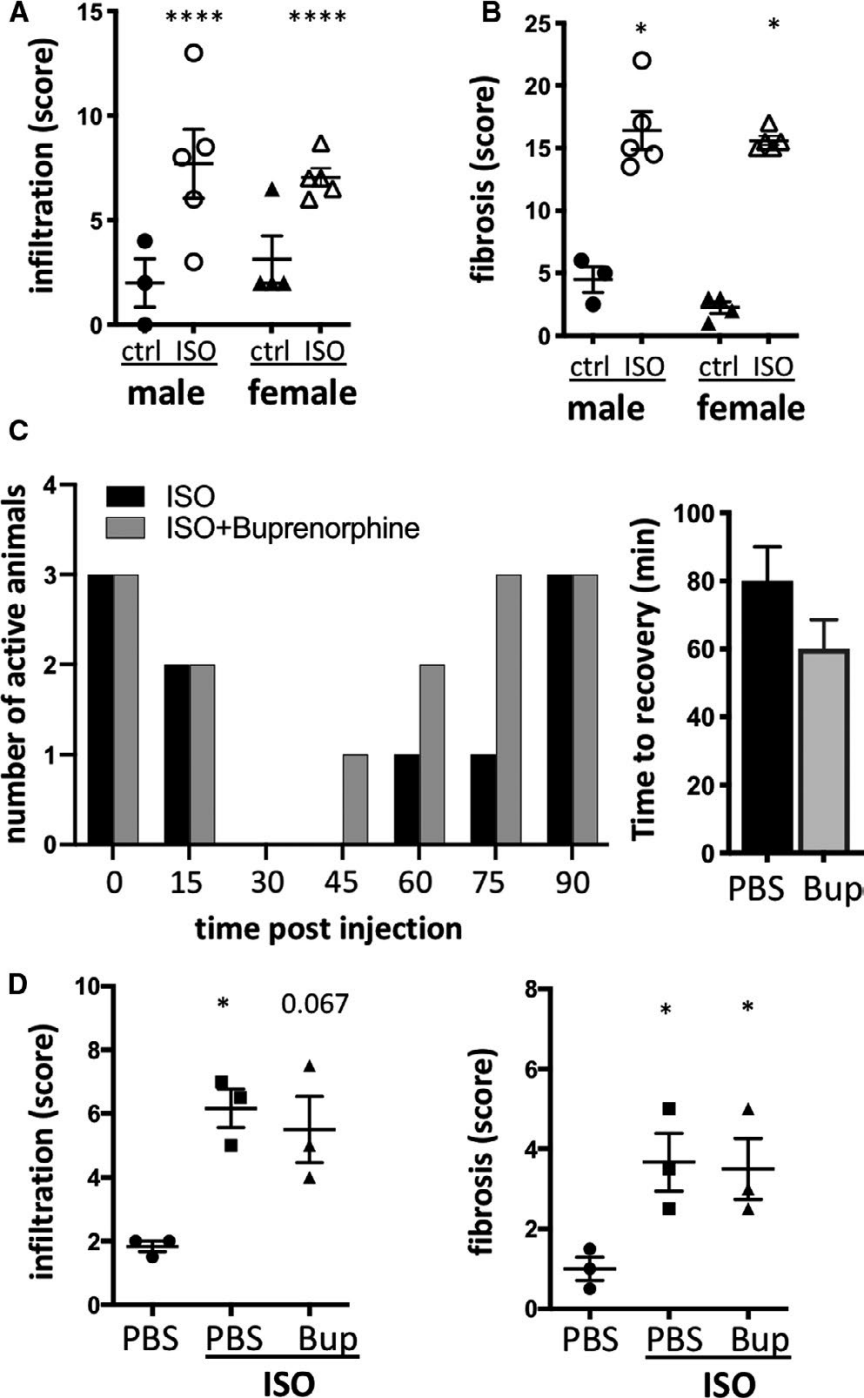
Opportunities for refinement and reduction in the isoproterenol‐induced T2MI model. Male and female C57BL/6J mice were treated with a single dose of isoproterenol to induce myocardial ischaemia and cardiomyocyte necrosis. A, B, Myocardial infiltration (A) and fibrosis (B) were scored on a scale from 0 to 3 (none, mild, moderate, severe) in 4 fields of view in 2 heart cross sections at mid‐level per mouse. n = 3‐5/group, symbols represent individual mice. C, D, Female C57BL/6J mice were treated with a single dose of isoproterenol with and without 0.05mg/kg buprenorphine to induce myocardial ischaemia and cardiomyocyte necrosis under analgesia. C, Count of animals with normal movement and activity and average time until recovery of normal activity pattern. D, Infiltration and fibrosis in cardiac tissue sections 2 weeks after isoproterenol injection. n = 3/group, symbols represent individual mice. Histopathology scoring data are expressed as mean ± s.e.m., *P < .05 (A, B: two‐way ANOVA with Sidak's multiple comparison post hoc test comparing each group to each other. D: non‐parametric Kruskal‐Wallis with Dunn's multiple comparisons post hoc test comparing each time‐point to baseline)

By convention, our first set of experiments was performed in male mice. However, considering a female dominance in autoimmune disease, immunological research is often performed in female mice. To reduce the number of surplus animals and ensure relevance to both sexes,^44^ we compared histology in isoproterenol‐injected male and female mice. Results showed an equivalent level of cardiac histopathology (Figure 4A, B) which allowed the use of female mice in subsequent immunological experiments and will also support the use of both sexes in the future.

To further improve animal welfare benefits of the isoproterenol model, we included pre‐treatment with 0.05mg/kg buprenorphine for pain relief. Buprenorphine was injected subcutaneously 30 minutes before isoproterenol administration. Manual mouse grimace scale (MGS) scoring was deemed not suitable, as no orbital tightening, or changes in nose and cheek bulge and whisker position were notable.^45^ Instead, we measured activity (moving, feeding, drinking, grooming) as a reliable sign of both the onset of and the recovery from acute isoproterenol effects (Figure 4C). Adding buprenorphine to the isoproterenol treatment shortened the average time to recovery by 20 minutes without affecting the degree of cardiac inflammatory and fibrotic damage (Figure 4D).

### 3.5 A single high dose of isoproterenol triggers dendritic cell (DC) accumulation in the heart

**Figure 5:**
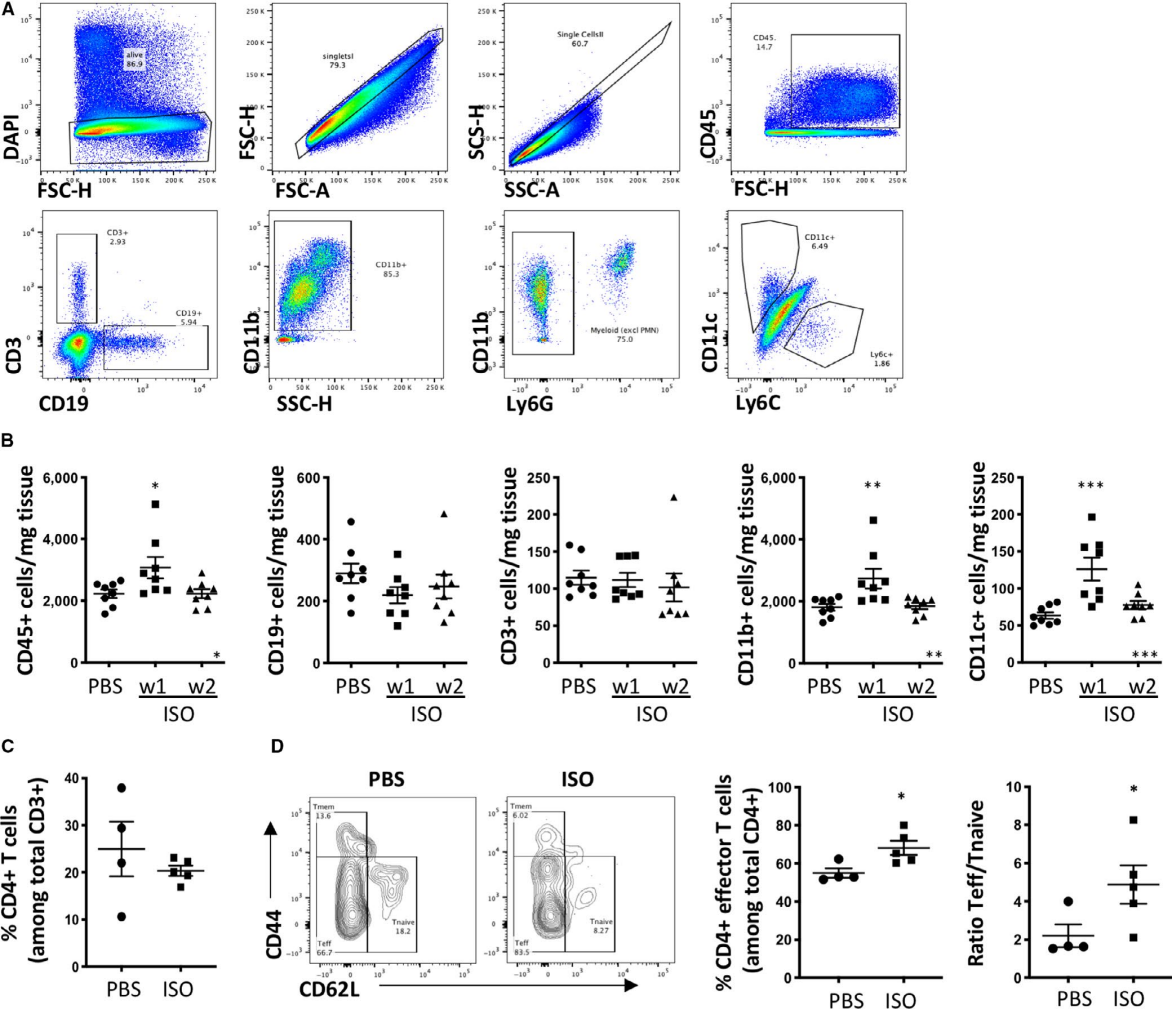
Myocardial CD45+ immune cell infiltrate in response to a single dose of 160mg/kg isoproterenol. Female C57BL/6J mice were treated with a single dose of isoproterenol to induce myocardial ischaemia and cardiomyocyte necrosis, and flow cytometry was performed on single‐cell preparations of heart ventricles 1 and 2 weeks after isoproterenol injection. A, Representative dot plots showing the flow cytometry gating strategy to obtain total CD45+, CD3+, CD19+, CD11b+ and CD11c+ populations. B, Quantification of the total number of CD45+, CD3+, CD19+, CD11b+ and CD11c+ cells per mg ventricular tissue in isoproterenol‐treated versus control mice. n = 8/group, symbols represent individual mice. C, Quantification of the fraction of CD4+ T cells among total T cells in the ventricular tissue of isoproterenol‐treated versus control mice. D, Representative contour plots of CD62L and CD44 staining of CD3+ CD4+ T cells and corresponding quantification of the CD62L‐CD44‐ subpopulation of effector T cells in isoproterenol‐treated versus control mice. n = 4‐5/group, symbols represent individual mice. Data are expressed as mean ± s.e.m., *P < .001, **P < .05, ****P < .0001 (B: one‐way ANOVA with Dunnett's multiple comparisons post hoc test comparing each time‐point to baseline. C, D: one‐tailed unpaired Student's *t* test with Welch's correction)

Isoproterenol‐induced cardiomyocyte damage leads to necrotic cell death,^33^ a potent trigger of innate immune responses initiating a classical wound healing response to remove cellular debris and restore tissue integrity.^46^ To investigate the composition of the myocardial immune cell infiltrate, we performed flow cytometry on single‐cell suspensions of ventricular tissue 1 and 2 weeks after isoproterenol treatment (Figure 5A). As expected, the CD45+ myocardial immune cell population was increased 1 week after isoproterenol injection and was largely composed of myeloid cells, whereas total numbers of adaptive CD19+ B and CD3+ T lymphocytes remained stable (Figure 5B).

Dendritic cells are crucial in the induction of adaptive immune responses and autoimmunity because of their unique ability of activating antigen‐specific CD4+ helper T cells.^47^ Their numbers peak 1 week after ischaemic damage induced by surgical LAD ligation in mice.^48^ Numbers of CD11c+ DC were significantly increased after 1 week in isoproterenol‐treated hearts, confirming this timing in the isoproterenol model. Importantly, increased DC number is the first step towards the induction of an adaptive immune response and autoimmunity against the heart.

### 3.6 Myocardial CD4+ helper T cells are activated in response to a single high dose of isoproterenol

To investigate whether early DC activation in response to isoproterenol‐induced cardiomyocyte necrosis is sufficient to trigger a downstream adaptive immune response, we performed a refined analysis of the myocardial CD4+ T cell phenotype by staining for the surface markers CD44 and CD62L which allow distinction between effector and naïve T cells. Although the total fraction of CD4+ T cells remained unchanged (Figure 5C), an increase in activated CD62L‐CD44‐ effector cells is apparent (Figure 5D). We also find activated CD4+ T cells in the mediastinal lymph nodes of male mice (Figure S2) showing relevance of these processes for both sexes. This confirms persistent activation of the adaptive immune system in response to isoproterenol‐induced myocardial injury.

### 3.7 Isoproterenol treatment triggers the production and in vivo deposition of mature anti‐heart auto‐antibodies

**Figure 6:**
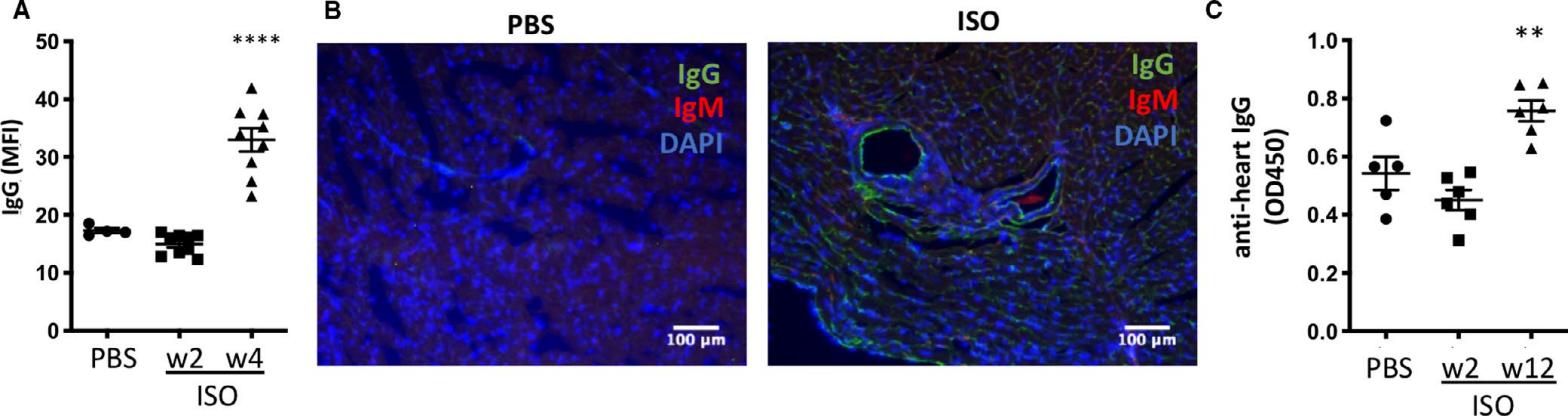
A single dose of isoproterenol triggers production of anti‐heart auto‐antibodies. Female C57BL/6J mice were treated with a single dose of 160mg/kg isoproterenol to induce myocardial ischaemia and cardiomyocyte necrosis, and the heart and serum were obtained for analysis of anti‐heart auto‐antibody production. A, Quantification of mean fluorescence intensity (MFI). B, Example image of immunofluorescence staining of frozen heart sections of isoproterenol‐treated mice 4 weeks after challenge, using goat anti‐mouse IgG‐FITC and rat anti‐mouse IgM‐PE to detect in vivo deposits of anti‐heart auto‐antibodies, green: IgG, red: IgM, blue: DAPI (nuclei). n = 4‐9/group. C, Levels of anti‐cardiac auto‐antibodies in the serum over time as tested by ELISA using serum of isoproterenol‐treated mice against rat cardiac lysate. n = 5‐6/group, symbols represent individual mice. Data are expressed as mean ± s.e.m., **P < .001, ****P < .0001 (one‐way ANOVA with Dunnett's multiple comparisons post hoc test comparing each time‐point to baseline)

Activated autoreactive CD4+ helper T cells induce B cells to generate mature auto‐antibodies, which are a hallmark sign of an established autoimmune response. We found a significant amount of mature IgG deposited in the myocardium 4 weeks after isoproterenol injection (Figure 6A, B). The linear deposition pattern followed the myocyte fibres and the inner lining of blood vessels. In particular, vessels surrounded by perivascular infiltrate appeared affected. In addition, a small number of IgG+ cells, presumably mature B cells, were found in both baseline and isoproterenol‐treated tissue. We further tested post‐isoproterenol serum at week 2 (acute) and week 12 (chronic) for the persistent presence of anti‐heart auto‐antibodies. We found a significant increase in auto‐antibody levels at chronic stage after isoproterenol injection (Figure 6C). Importantly, these auto‐antibodies are of the mature class‐switched IgG isotype as present in established and persistent adaptive immune responses.

This confirms that mature autoreactive B cells persist for an extended period after initial damage and produce class‐switched auto‐antibodies against the heart, which deposit on cardiomyocytes and endothelial cells in vivo, which is the prerequisite for auto‐antibody–mediated tissue damage.

### 3.8 Adoptive transfer of splenocytes from isoproterenol‐treated C57BL/6J mice is sufficient to induce LV dilation and functional decline

Adoptive transfer of autoreactive immune cells is a well‐established strategy to prove their pathological effects isolated from potential confounding factors because of the initial experimental challenge. A recent report shows that splenic T cells isolated from C57BL/6 mice subjected to permanent coronary ligation induce LV remodelling upon transfer into healthy recipient mice.^49^

**Figure 7:**
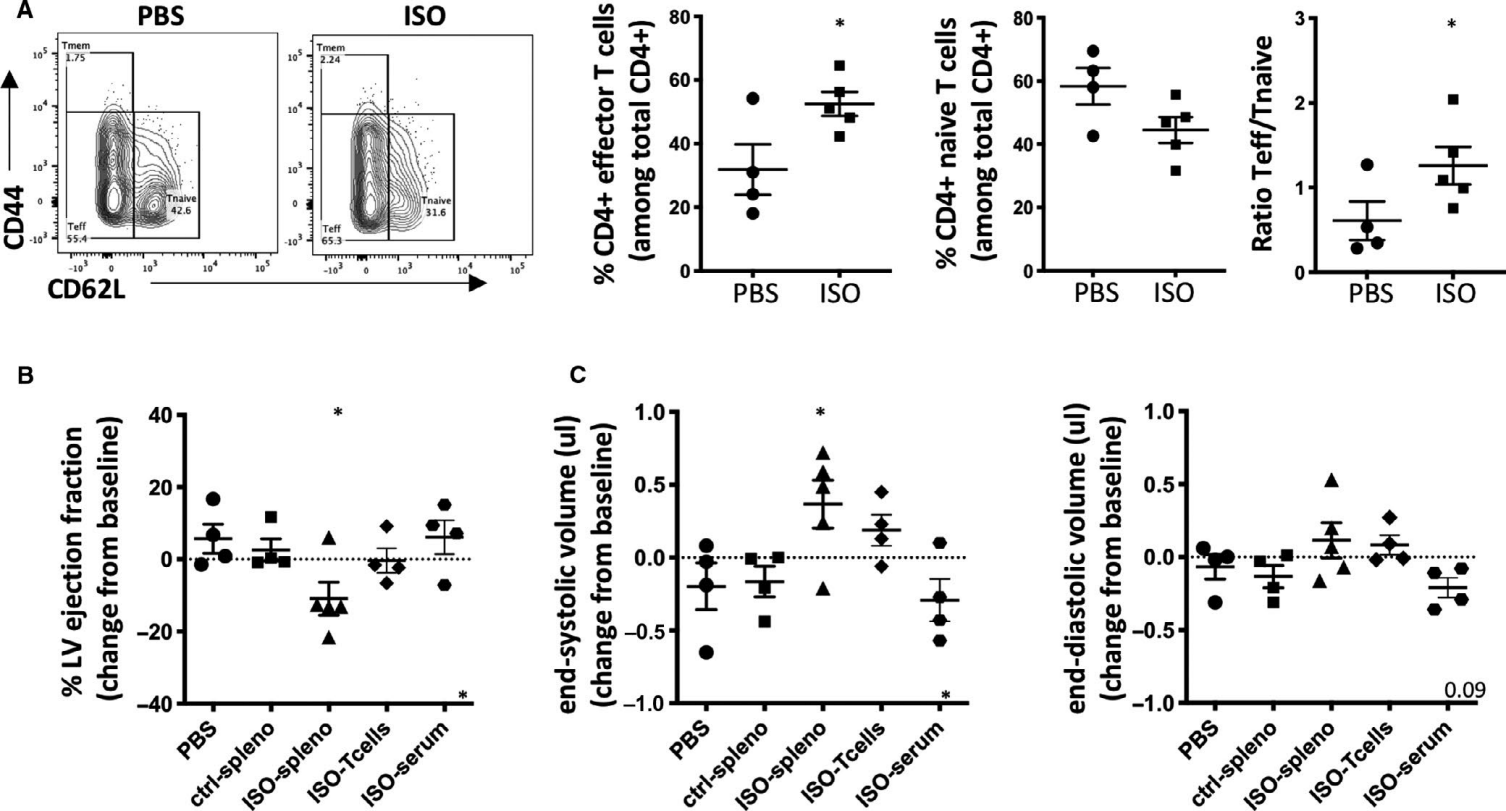
Adoptive transfer of splenocytes from isoproterenol‐injected donor mice leads to LV dilation and impairs cardiac function. Female donor C57BL/6J mice were treated with a single dose of isoproterenol to induce myocardial ischaemia and cardiomyocyte necrosis. Serum was collected and splenocytes and CD4+ T cells isolated after 4 weeks. A, Representative contour plots of CD62L and CD44 staining of splenic CD3+ CD4+ T cells and corresponding quantification of the CD62L‐CD44‐ subpopulation of effector T cells in isoproterenol‐treated versus control mice. n = 4‐5/group, symbols represent individual mice. Recipient mice were treated with an intravenous injection of 3x10^7^ control (ctrl‐spleno) or ISO‐splenocytes (ISO‐spleno), 1x10^7^ ISO‐T cells or a twice‐weekly intraperitoneal injection of 200ul ISO‐serum for 2 weeks. B, C, Echocardiography to assess global LV function (LV ejection fraction, B) and heart morphology (end‐systolic and end‐diastolic volumes, C) was performed after 4 weeks. Data are expressed as mean ± s.e.m., *P < .001, **P < .05, ****P < .0001 (A: one‐tailed unpaired Student's t test with Welch's correction. B, C: one‐way ANOVA with Dunnett's multiple comparisons post hoc test comparing each group to ctrl‐spleen)

To show whether immunopathology is also transferable in isoproterenol‐induced T2MI, we performed an adoptive transfer of total splenocytes, splenic CD4+ T cells (T cell–mediated damage) or serum (auto‐antibody mediated damage) from isoproterenol‐treated mice 4 weeks post‐injection, to healthy syngeneic recipients. Splenocytes were used because of the obtainable numbers and after confirmation that an increase in the CD44‐CD62L‐ effector subpopulation is also observed in splenic CD4+ T cells (Figure 7A).

A single intravenous transfer of 3x10^7^ splenocytes from isoproterenol‐treated mice affected global cardiac function as shown by a drop in LV ejection fraction 4 weeks after transfer (Figure 7B). This functional impairment was likely because of impaired contractility and dilation as LV volumes increased in recipients of isoproterenol‐splenocytes (Figure 7C). Notably, transfer of 1x10^7^ isolated CD4+ T cells only partially recapitulated the effects of total splenocytes and we did not observe any differences between control groups and mice treated with serum from isoproterenol‐injected donor mice (Figure 7B, C).

This confirms that besides acute myocardial necrosis and subsequent fibrotic tissue repair, isoproterenol injection triggers the development of long‐term autoimmunity capable of inducing heart damage and affecting cardiac function. This is likely a multi‐dimensional process caused by the interplay between several immune cells and factors.

## 4 DISCUSSION

**Figure 8:**
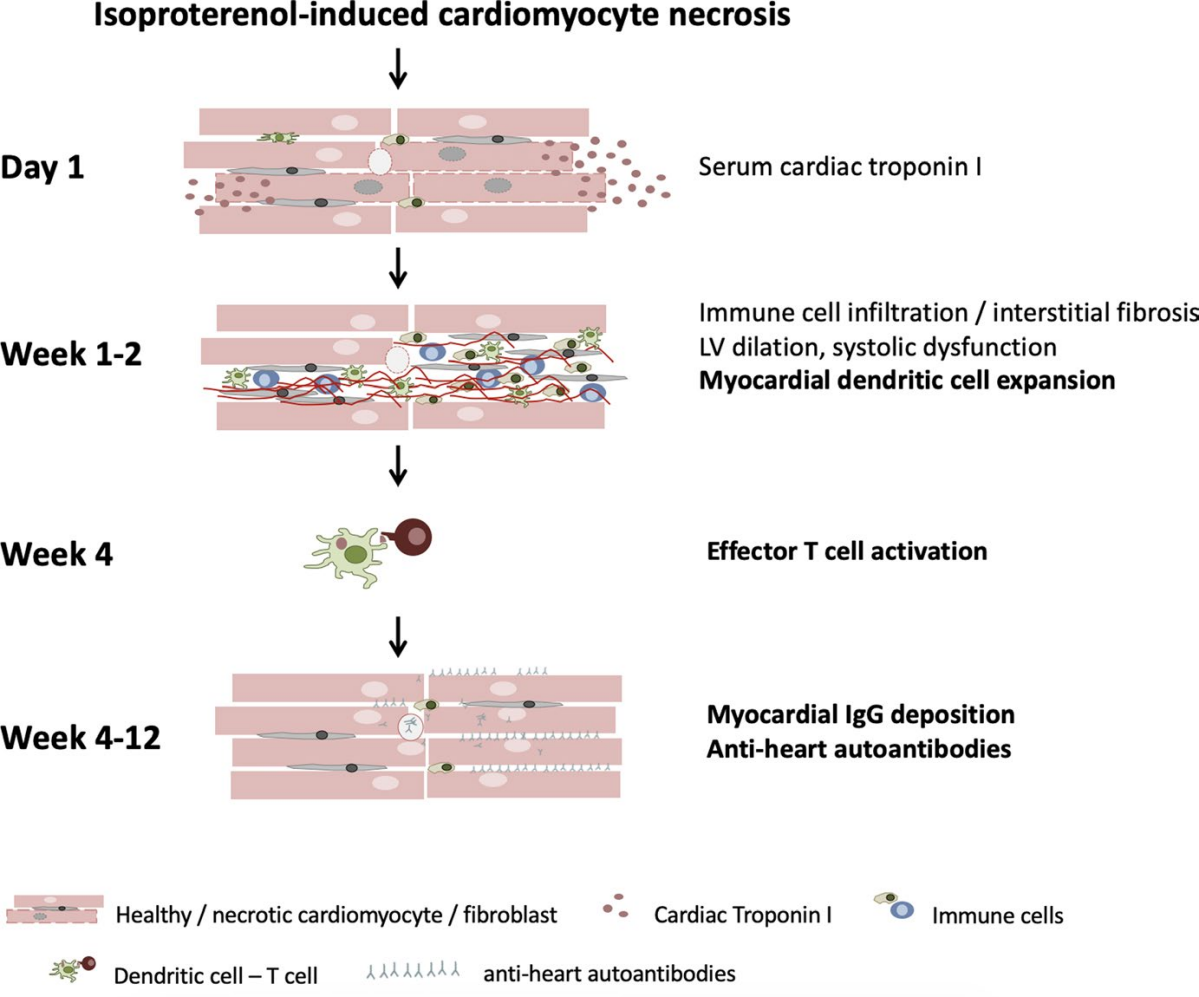
Isoproterenol‐induced cardiomyocyte necrosis leads to the passive release of alarmins and antigens including cardiac troponin I, which is used as serum biomarker for cardiac damage. Pro‐inflammatory alarmins trigger acute innate immune cell infiltration including DC at week 1 after isoproterenol challenge. Interstitial fibrosis and morphological and functional changes are also observed at this time. Myocardial DC activate auto‐reactive effector T cells in the mediastinal lymph nodes. Effector T cells in turn induce B cells to generate mature auto‐antibodies that deposit along myocardial fibers.

Here, we show that T2MI‐like necrotic lesions induced by a single high dose of the synthetic catecholamine isoproterenol trigger persistent adaptive immunopathology in the heart (Figure 8)

The definition of myocardial infarction differentiates patients with MI because of coronary occlusion and plaque rupture (T1MI) from those with myocardial necrosis because of oxygen supply‐demand imbalance (T2MI).^50^, ^51^ Myocardial necrosis without symptoms or signs of myocardial ischaemia is classified as acute or chronic myocardial injury. Both myocardial injury and T2MI are common, yet these patients have poor short‐term and long‐term outcome ^7^, ^51^ with a heart failure risk comparable to T1MI.^8^ Injection of a single high dose of isoproterenol mimics an excessive activation of the adrenergic system and leads to sympathetic overstimulation of the heart. This can be observed during critical illness in human patients ^52^ including those with takotsubo syndrome ^53^, ^54^ and in septic patients ^55^ or those receiving catecholamines for cardiac pacing or inotropic support. Detrimental effects of excess catecholamines on the heart include impaired diastolic function, tachycardia and tachyarrhythmia.^52^ The consequent imbalance between myocardial oxygen supply and demand, dysregulation of calcium handling and the resulting degree and pattern of myocardial necrosis, inflammatory infiltration and replacement fibrosis are reminiscent of the myocardial damage incurred by human T2MI. Notably, the autonomous nervous system and aberrant adrenergic stimulation of the heart have also been suggested as a clinically targetable pathological factor after myocardial infarction ^56^ and in the development of heart failure.^57^, ^58^

Concerns about off‐target damage to other organs have previously hampered acceptance of the isoproterenol model. Using our treatment regimen, these appear uncalled‐for as no direct isoproterenol‐induced pathological changes were observed in liver, lung and skeletal muscle. Damage to the kidneys was of particular interest as there are a range of isoproterenol‐activated pathways that may sensibly be assumed to cause kidney injury. For example, isoproterenol‐induced activation of the renin‐angiotensin cascade may lead to liberation of angiotensin II,^59^ which can cause renal vasoconstriction driving ischaemia and fluid overload and promotes renal oxidative stress, inflammation and fibrosis.^60^ However, because of belated onset, the mild acute tubular injury and fibrosis detected in the kidneys 2 weeks after treatment are most consistent with cardiorenal syndrome, a positive feedback loop in which a damaged heart leads to renally mediated fluid retention, which then further aggravates the underlying heart dysfunction.^42^

The immune response following myocardial injury has received significant attention recently. It is initiated by the release of danger‐associated molecular patterns (DAMPs) from necrotic cardiomyocytes, which activates a highly orchestrated innate immune reaction ^46^ for the quick removal of necrotic debris and restoration of tissue integrity. The interplay between innate immune cells and fibroblast plays a crucial role in the ensuing scar formation. Although this immediate wound healing response is critical for acute survival, the heart appears susceptible to persistent immunopathology presumably because of an adaptive autoimmune response against cardiac antigens including most prominently cardiac myosin.^61^ This anti‐heart autoimmune response has recently become the focus of studies into how the immune system affects the heart after ischaemic damage. Both autoreactive B and T cells have been detected after myocardial damage in mouse models ^62^, ^63^ and human patients.^64^, ^65^

We show here that isoproterenol‐induced myocardial necrosis triggers dendritic cell expansion in the myocardium and T cell activation in the mediastinal lymph nodes. Activated T cells stimulate B cells to produce mature IgG anti‐heart auto‐antibodies against the heart, a sign of a bona fide autoimmune response. Importantly, we confirm cell‐mediated immunopathology against the heart by adoptive transfer of splenocytes from T2MI mice.

Anti‐heart auto‐antibodies indicating autoimmunity have thus far been demonstrated in a wide range of human heart conditions ^66^ and are likely because of high amounts of cardiac antigen in an inflammatory environment overwhelming immunological tolerance. Importantly, the adaptive immune system is activated by antigens released from necrotic cells irrespective of the initial cause of necrosis. Accordingly, we found anti‐heart auto‐antibodies in experimental models after surgical induction of T1MI via LAD ligation ^16^ and under systemic inflammatory conditions,^30^ as well as in the present study after chemical induction of T2MI‐like myocardial necrosis. The common factor in these situations is myocardial necrosis, which is sufficient to induce a downstream adaptive immune response against the heart.

Autoimmunity against the heart causes persistent myocardial immunopathology and ongoing tissue degeneration, which exacerbates development towards heart failure.^67^ Underlying pathological mechanisms are still far from understood, but the isoproterenol model provides an opportunity to study these processes in T2MI‐like heart disease. Simplicity and resource efficiency will allow thorough investigation of a range of aspects that likely affect short‐ and long‐term outcome in T2MI patients including investigation of (a) the local innate immune response in the myocardium, (b) the role of lymphatics and fibroblasts in post‐T2MI autoimmunity,^68^, ^69^ (c) the influence of genetics on the risk of developing heart failure after T2MI ^34^ and (d) how all of the above can be targeted for improved therapeutics.

### 4.1 Study limitations

Immune cells express adrenoreceptors,^70^, ^71^ and long‐term isoproterenol treatment regimens increase circulating immune cells.^72^ Because of an in vivo half‐life of less than 5 minutes, the effects of a single acute isoproterenol injection are expected to have worn off by the time cardiomyocytes develop necrosis and the immune system is recruited.^73^ However, an effect on the early immune response cannot be fully excluded and should be considered when studying very early responses.^74^

Our adoptive transfer experiment indicates a role of CD4+ T cells in cardiac immunopathology, but as expected they are not the sole pathogenic factor. Serum transfer (to test the role of circulating auto‐antibodies) failed to induce a cardiac phenotype which may have several technical (eg insufficient amount of serum transferred) and/or biological reasons (e.g. majority of anti‐heart auto‐antibodies are bound to donor myocardium, 4 weeks are not enough to show pathology or isolated auto‐antibodies are not pathological). Further studies are therefore needed for conclusive information on which cell type and/or factor has true pathological function in driving LV dilation and heart failure. Heart failure is a complex condition, and it is anticipated that the majority of immune cells will be involved.

This study did not investigate the effects of isoproterenol on the brain. Researchers wishing to address the neurological aspects of T2MI are encouraged to first investigate off‐target brain histopathology and damage markers.

The degree of isoproterenol‐induced immunopathology and replacement fibrosis, and other physiological effects of catecholamines are variable between mouse strains.^75^, ^76^ Although this opens exciting opportunities for genetic mapping of variable traits, we recommend performing a dose titration experiment for new strains. C57BL/6J mice have a robust cardiac phenotype and are relatively resistant to myocardial fibrosis.^34^, ^35^ It is anticipated that other strains may be more susceptible to isoproterenol effects and a dose titration experiment will be necessary to maintain welfare standards.

The decline of global cardiac function we observed in this study in C57BL/6J mice was significant but mild compared to clinical definitions of human heart failure both with reduced and preserved ejection fraction.^77^ Other mouse strains may react with a more pronounced functional phenotype.

The role of fibroblasts and other cardiac resident cell types has not been investigated in this study. However, the specific contribution of fibroblasts to histopathology may vary between isoproterenol‐induced T2MI and other types of cardiac damage such as LAD ligation‐induced T1MI or experimental autoimmune myocarditis and is worth exploring further.^78^

Several additional factors that might affect the response to isoproterenol treatment have not been investigated systematically including age and potential age‐dependent sex differences, circadian rhythm and seasonality, as well as holding conditions (e.g. SPF versus conventional animal facility affecting microbiome).

## CONFLICT OF INTEREST

The authors declare no conflict of interest.

## AUTHOR CONTRIBUTIONS

**Elvira Forte:** Conceptualization (equal); Formal analysis (equal); Methodology (equal); Validation (equal); Visualization (equal); Writing‐review & editing (equal). **Mona Panahi:** Investigation (equal); Visualization (equal). **Nicoleta Baxan:** Investigation (equal); formalAnalysis, methodology, validation, writing Original Draft. **Fu Siong Ng:** Formal analysis (equal); Writing‐original draft (equal); Writing‐review & editing (equal). **Jane Branca:** Investigation (equal). **Olivia Bedard:** Investigation (equal). **Muneer G. Hasham:** Investigation (equal). **Lindsay Benson:** Methodology (equal); Investigation (equal). **Joseph Boyle:** Formal analysis (equal); Writing‐review & editing (equal). **Sian E Harding:** Funding acquisition (equal); Project administration (equal); Resources (equal); Supervision (equal). **Nadia Rosenthal:** Funding acquisition (equal); Project administration (equal); Resources (equal); Supervision (equal). **Susanne Sattler:** Conceptualization (equal); Data curation (equal); Formal analysis (equal); Funding acquisition (equal); Investigation (equal); Methodology (equal); Project administration (equal); Resources (equal); Supervision (equal); Validation (equal); Visualization (equal); Writing‐original draft (lead); Writing‐review & editing (equal).

## Supporting information

Figure S1

Figure S2

## Data Availability

The data that support the findings of this study are available from the corresponding author upon reasonable request.
