# Supplementary material for: Type 2 MI induced by a single high dose of isoproterenol in C57BL/6J mice triggers a persistent adaptive immune response against the heart
Source: J Cell Mol Med. 2020 Nov 29;25(1):229–43. doi: 10.1111/jcmm.15937 (PMC7810962; doi:10.1111/jcmm.15937)

Supplementary information 1

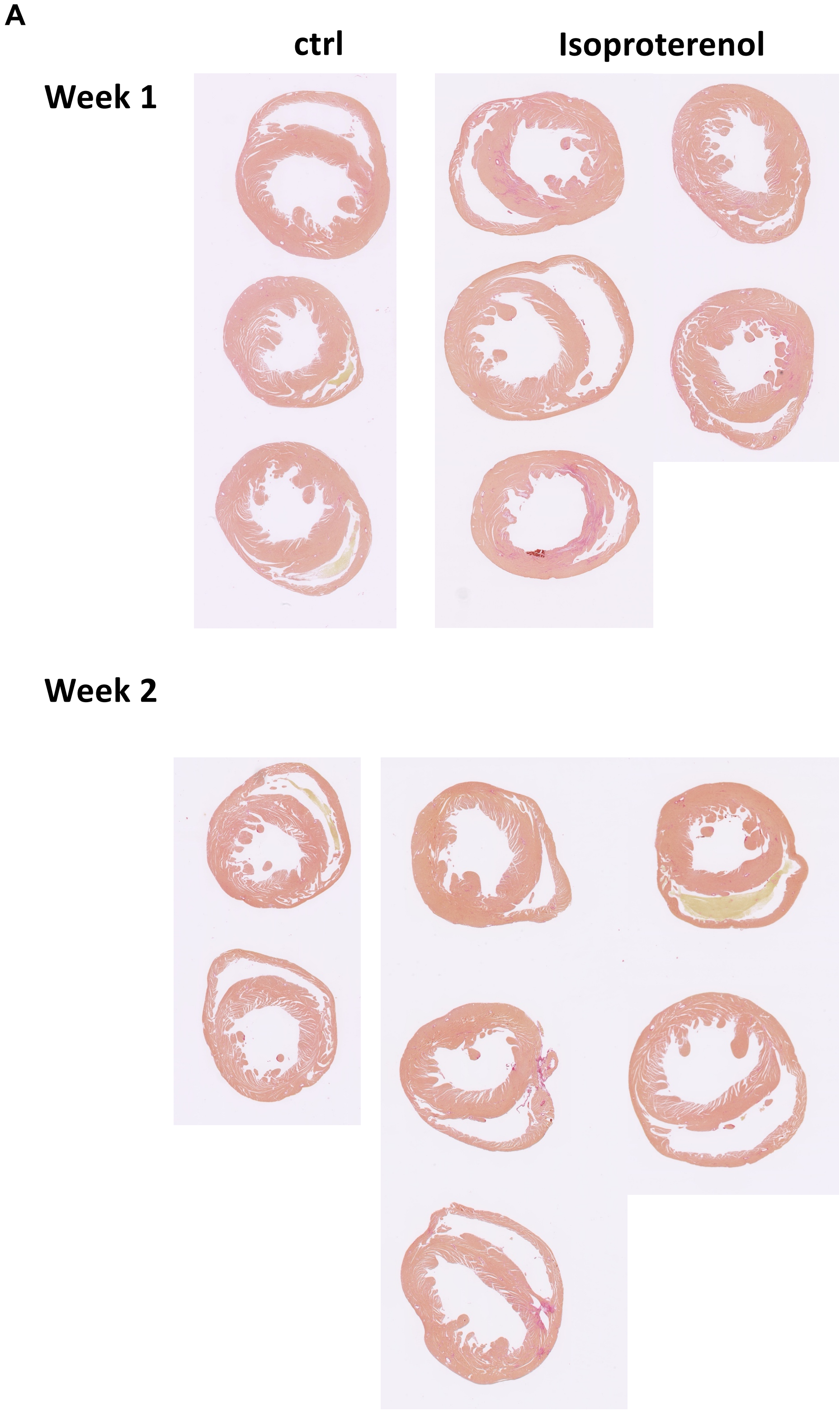

Figure S1

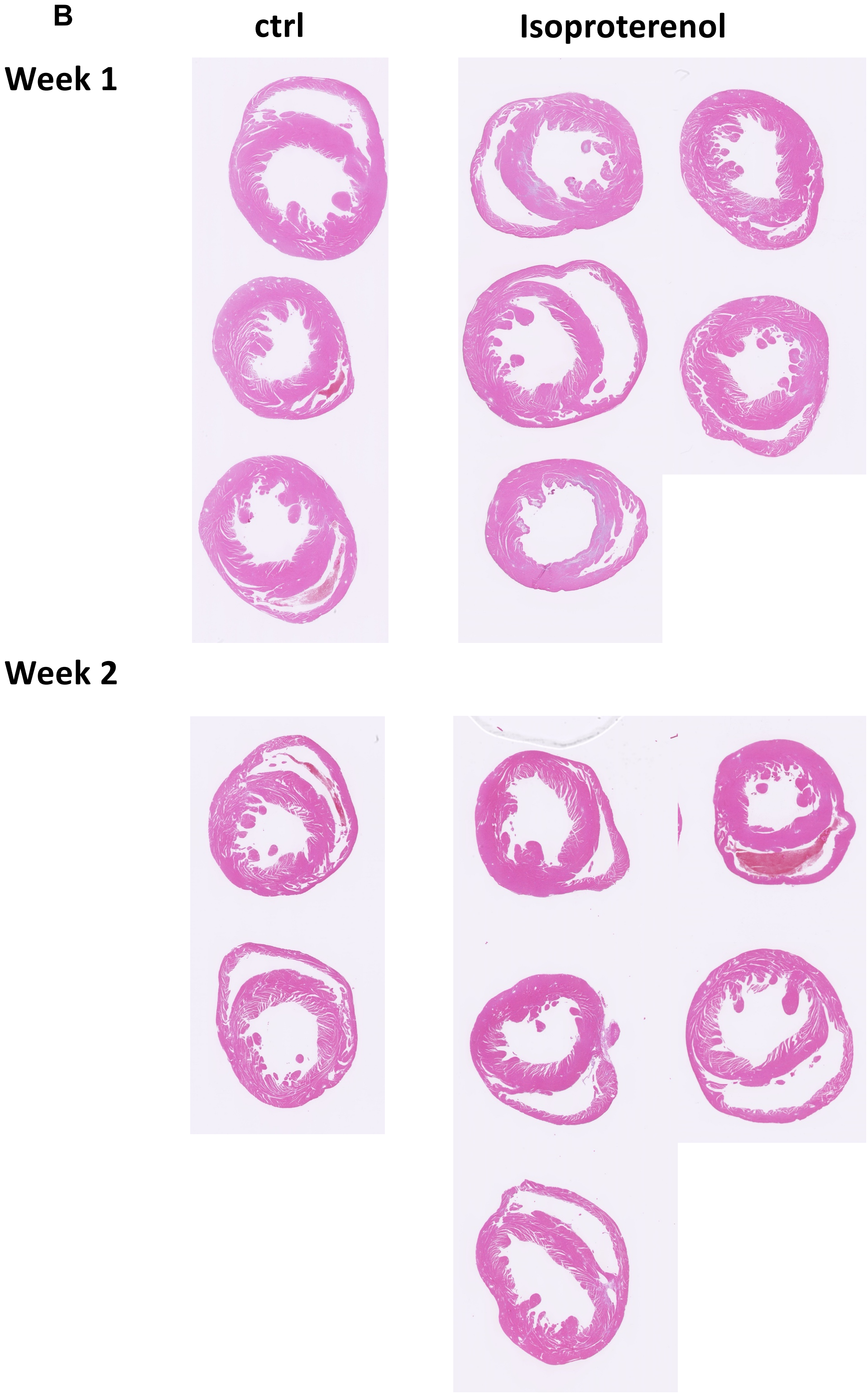

Figure S1

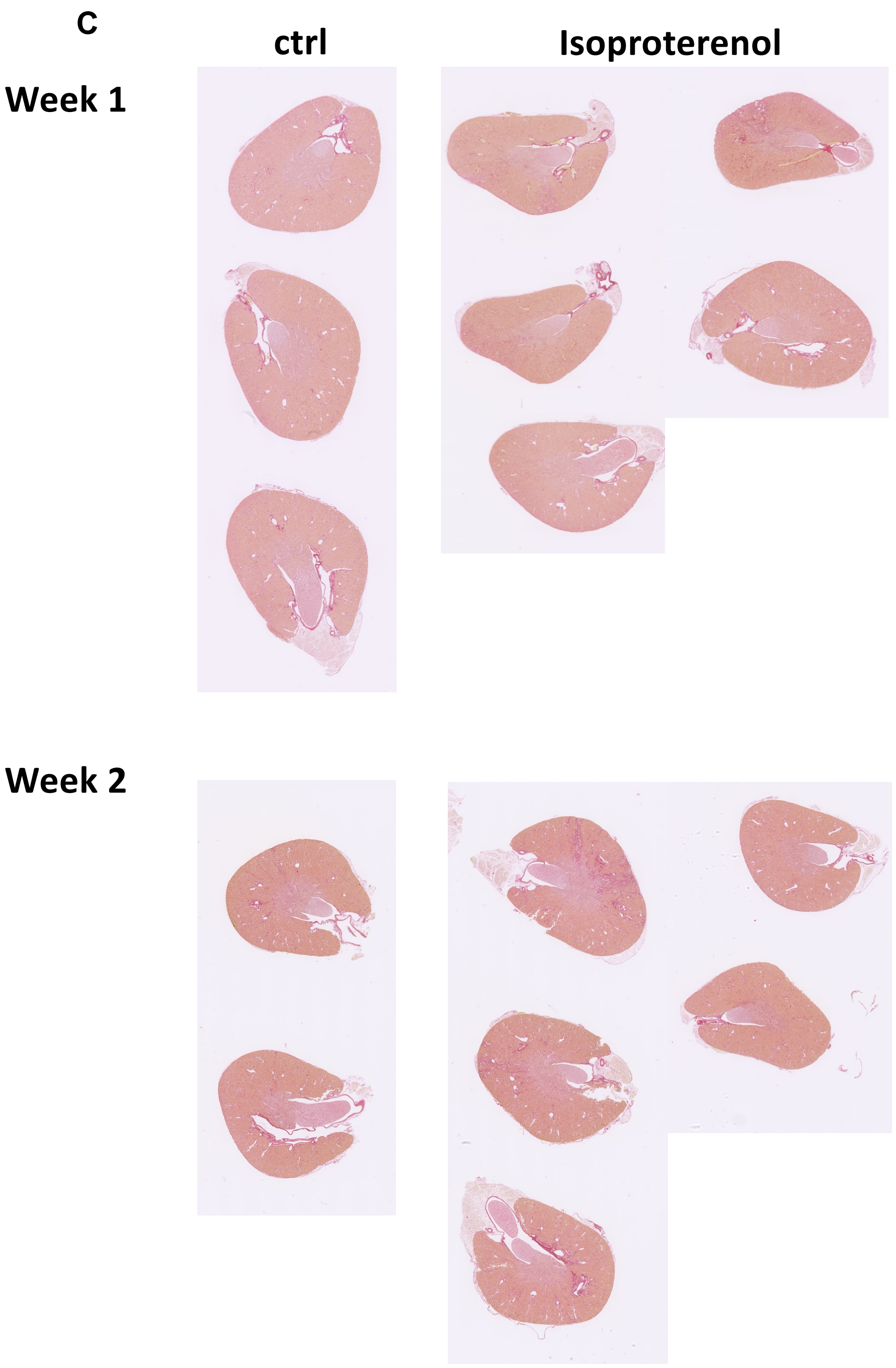

Figure S1

D

ctrl | Isoproterenol

Week 1

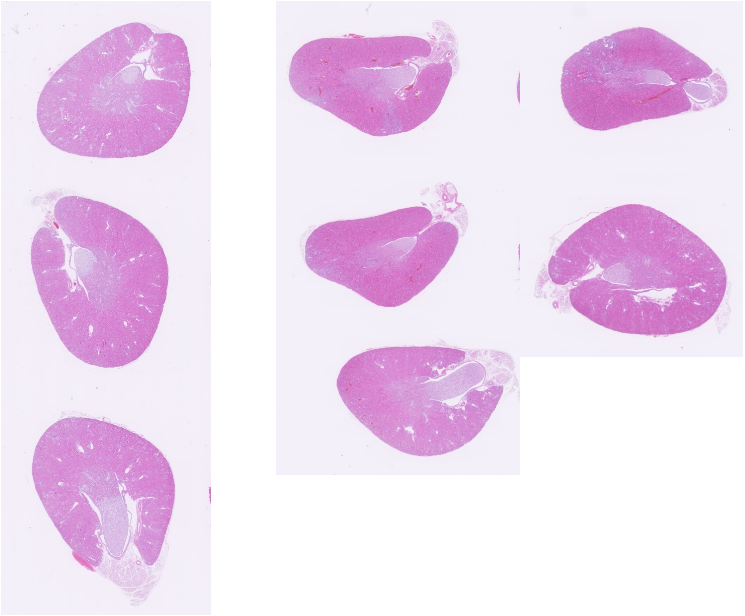

Week 2

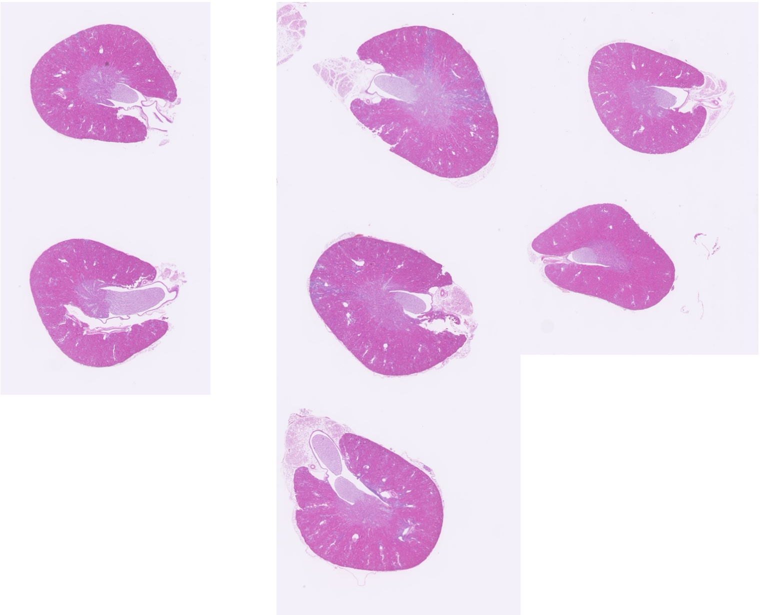

Figure S1

E

ctrl | Isoproterenol

Week 1

Week 2

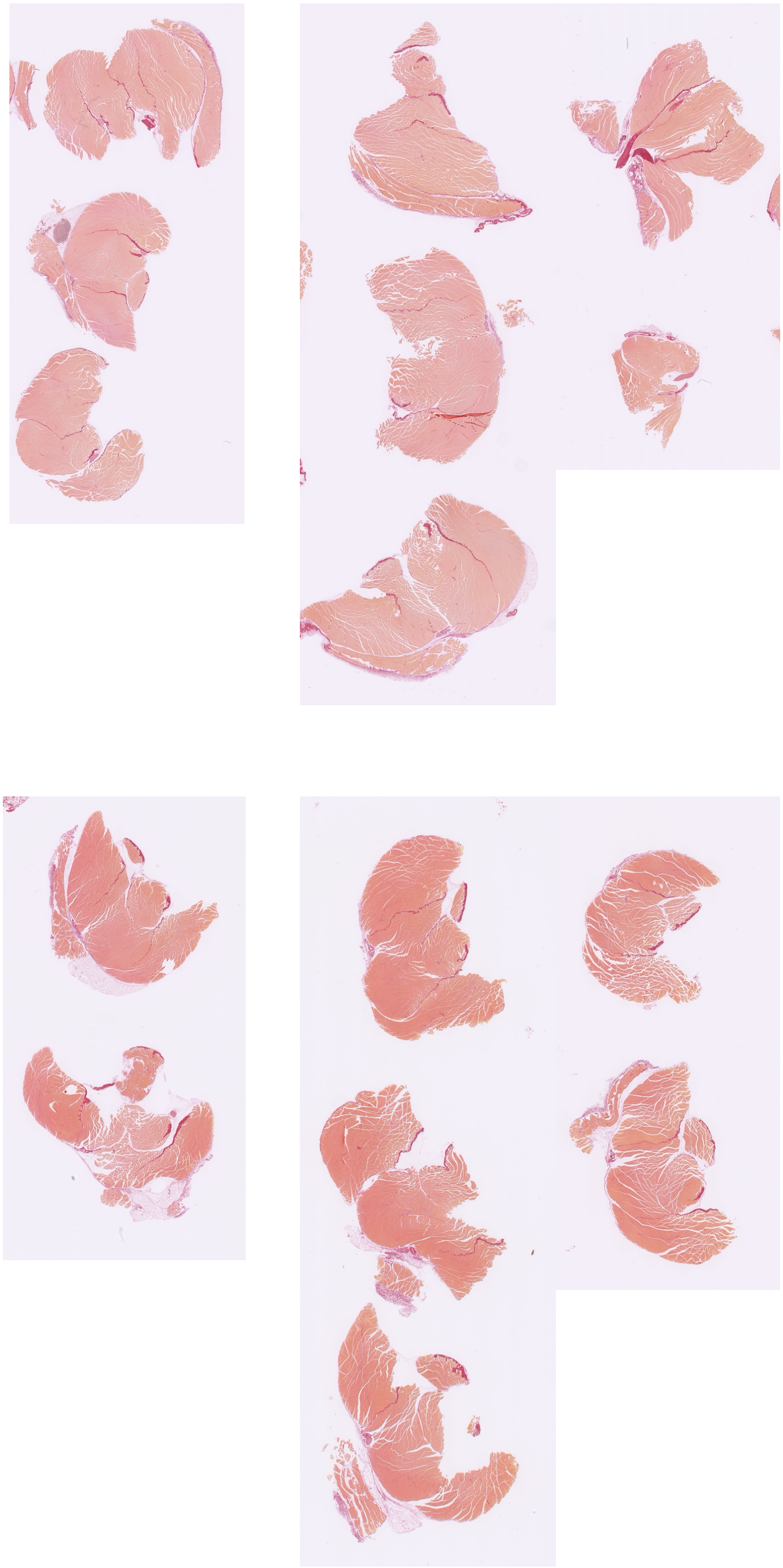

Figure S1

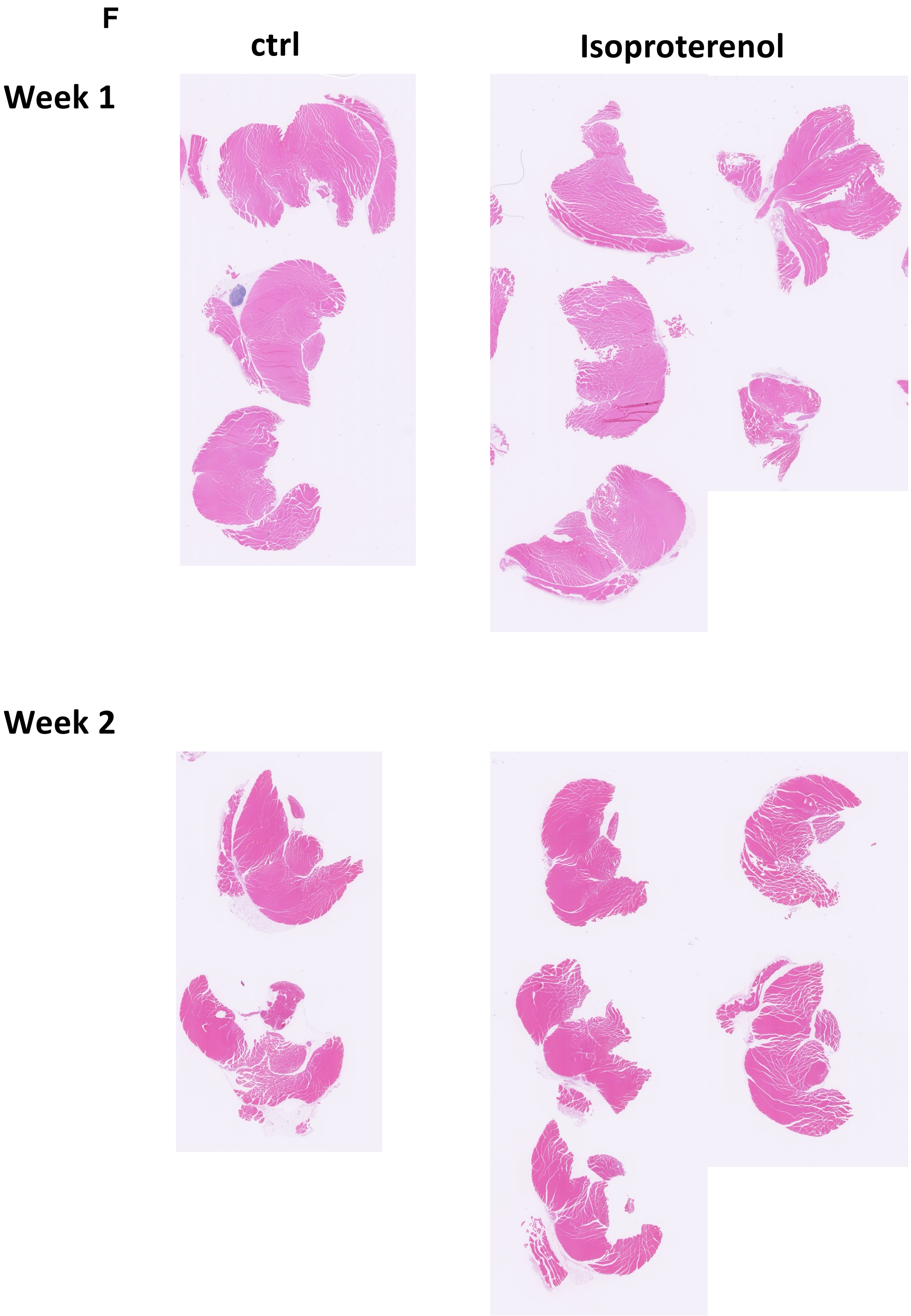

Figure S1

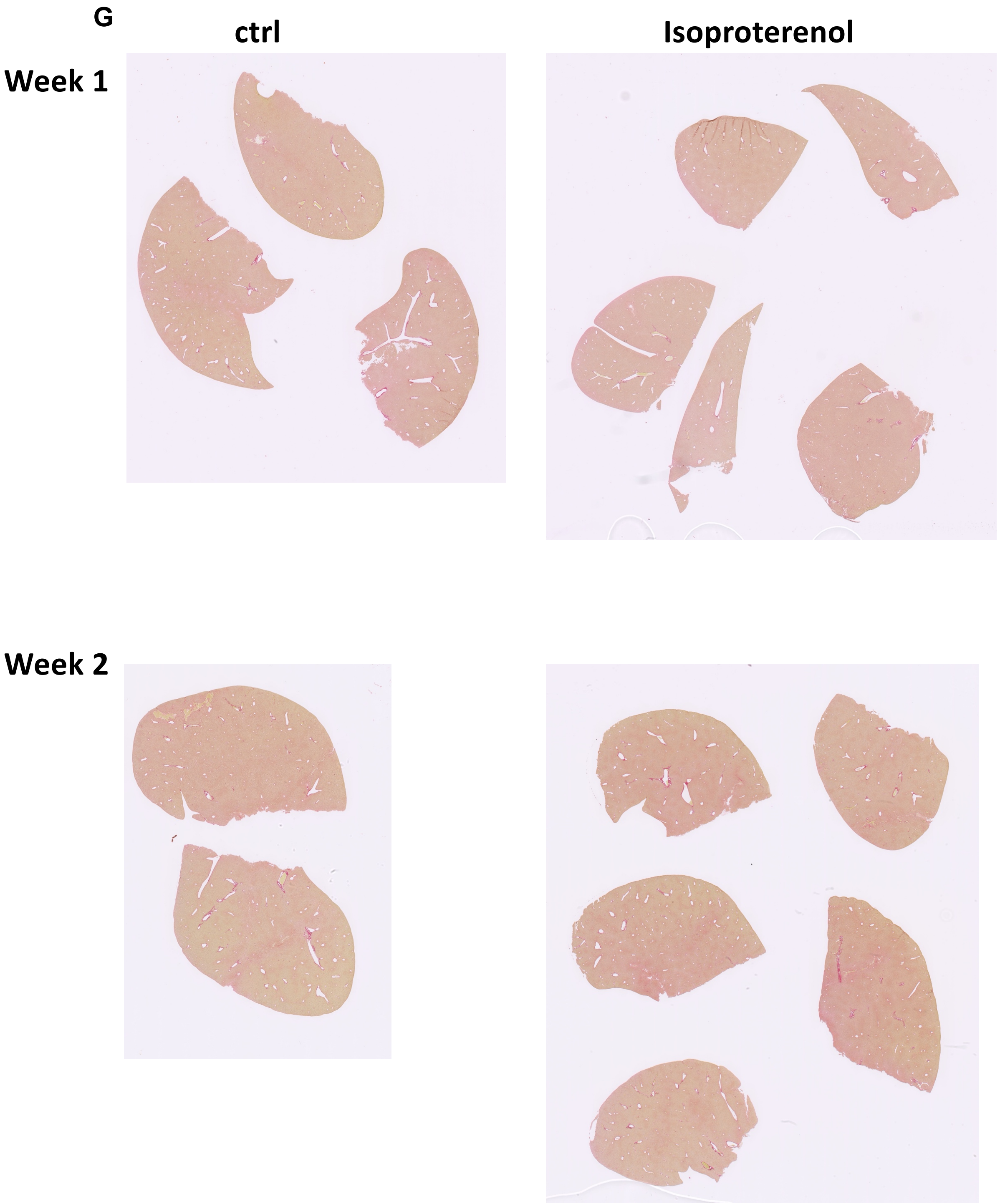

Figure S1

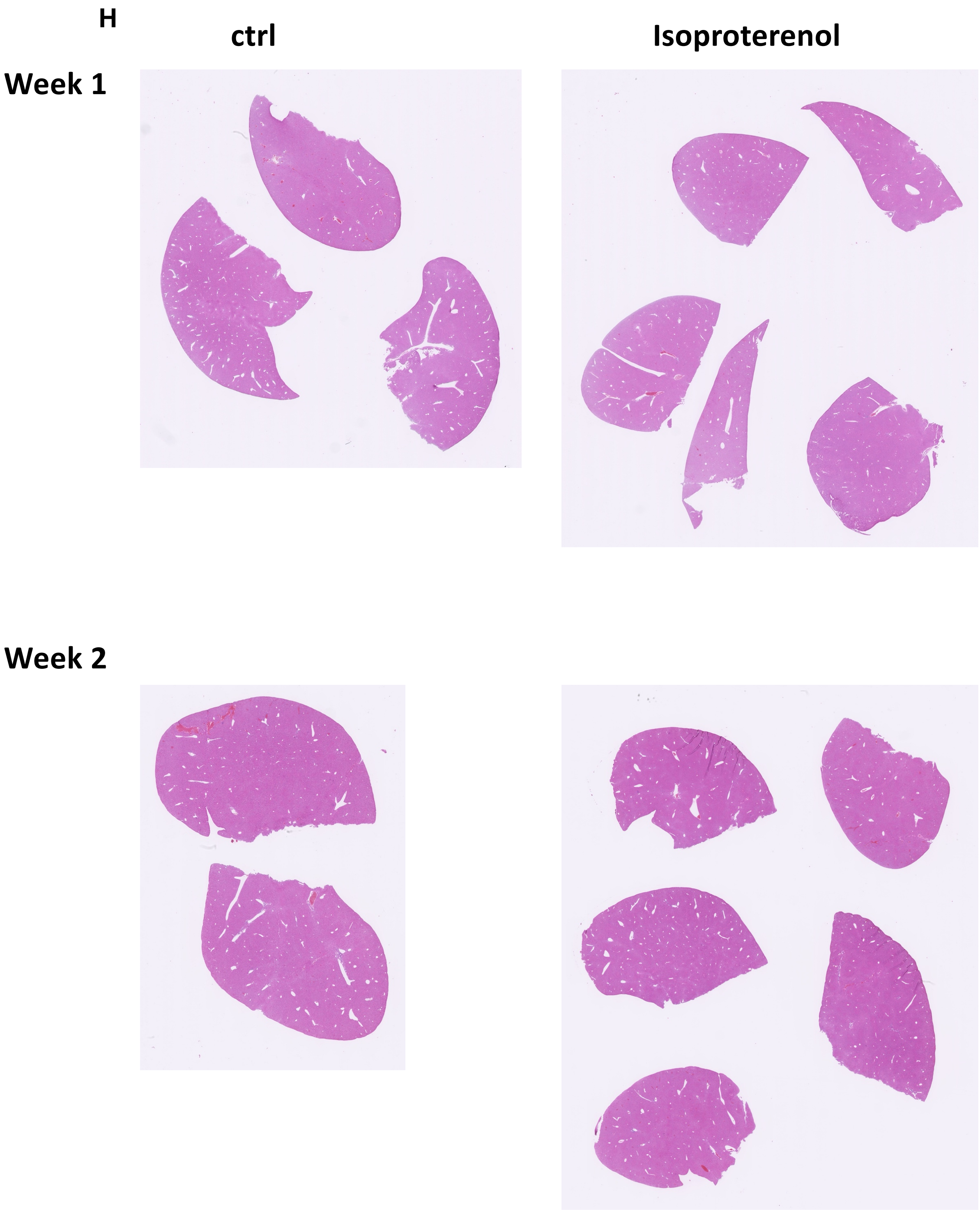

Figure S1

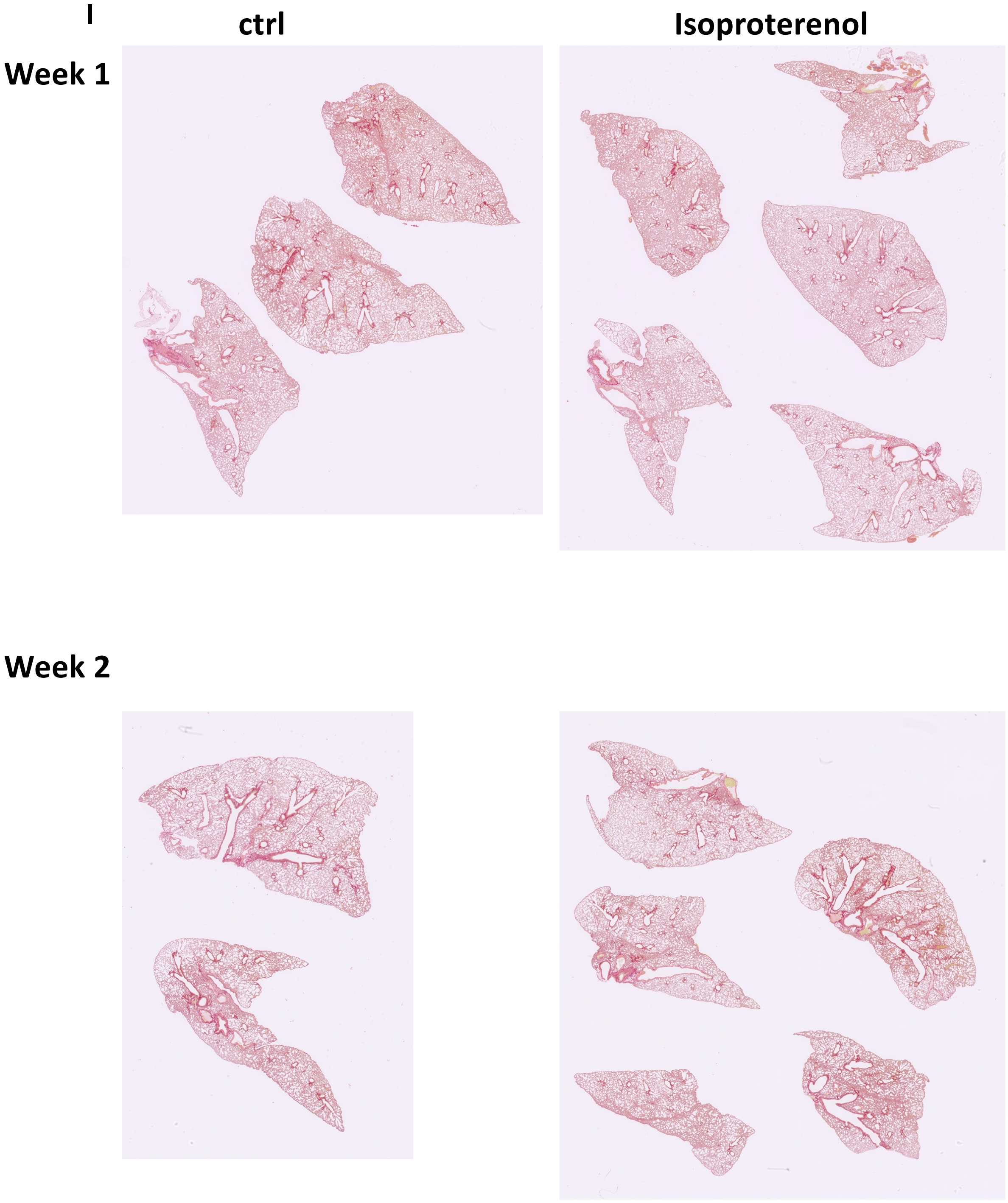

Figure S1

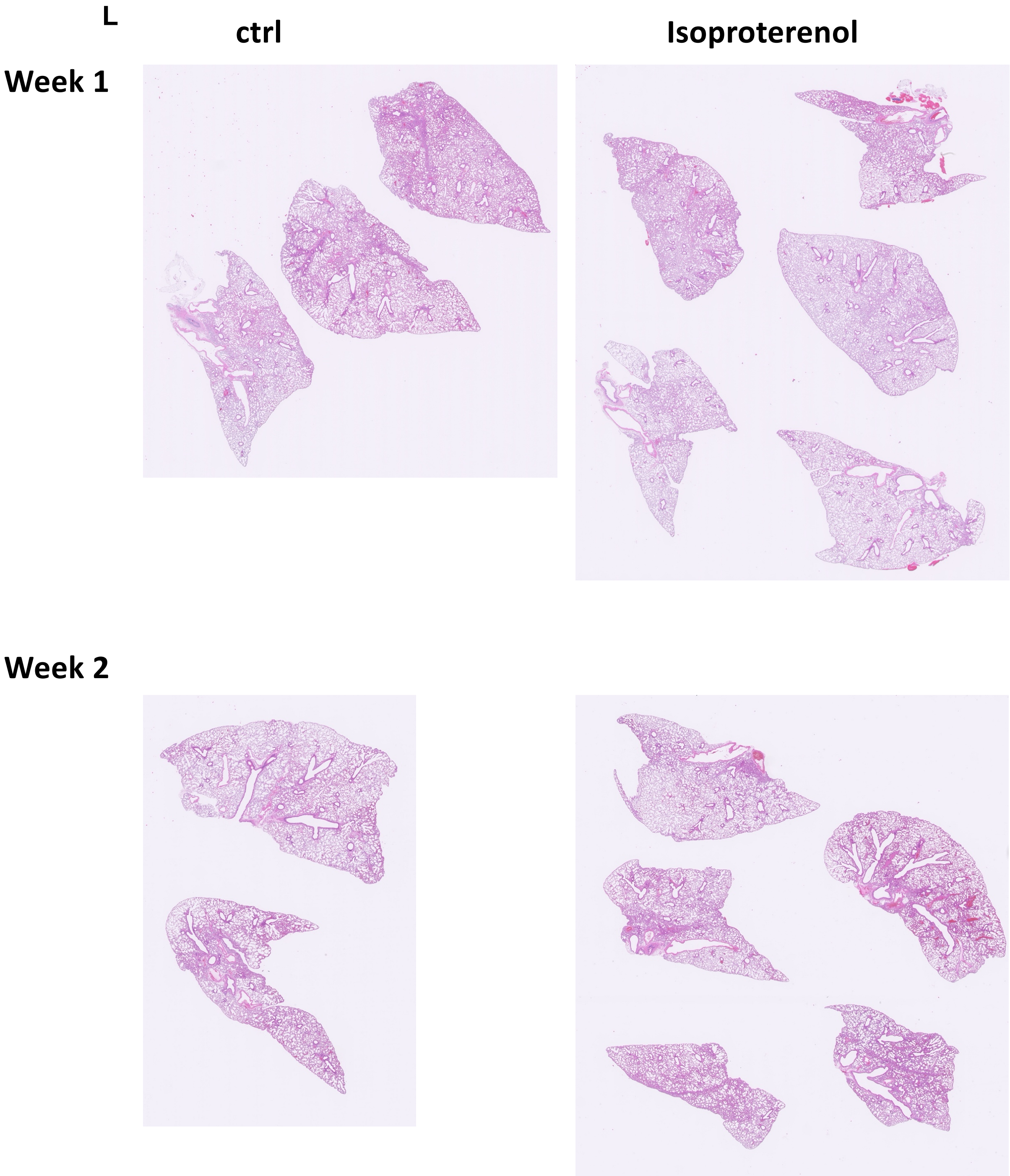

Supplement: Supplementary file 1 — Figure S1 [file JCMM-25-229-s001.pdf]
